# Viscoelastic nanomechanical devices for neuromorphic information processing

**DOI:** 10.1126/sciadv.aeg9893

**Published:** 2026-09-16

**Authors:** Peter F. Satterthwaite, Sarah O. Spector, Maxwell Conte, Teddy Hsieh, Eduard O. Bobylev, Srinidhi Venkatesh, Jeremiah A. Johnson, Farnaz Niroui

**Affiliations:** ^1^ Department of Electrical Engineering and Computer Science, Massachusetts Institute of Technology, Cambridge, MA 02139, USA.; ^2^ Research Laboratory of Electronics, Massachusetts Institute of Technology, Cambridge, MA 02139, USA.; ^3^ Department of Materials Science and Engineering, Massachusetts Institute of Technology, Cambridge, MA 02139, USA.; ^4^ Department of Chemistry, Massachusetts Institute of Technology, Cambridge, MA 02139, USA.

## Abstract

Information processing through intrinsic physical processes in materials enables energy-efficient, bioinspired computing paradigms. In particular, mechanical transformations at the nanoscale stand out as a versatile and efficient mechanism for sensitive and highly tunable control. Here, we introduce an in-material computing platform leveraging the mechanical properties of nanoscale soft matter. Overcoming the limitations of conventional mechanical systems, our platform enables subnanometer control and engineered dynamics, which we demonstrate in an electromechanically tunable tunneling junction composed of a nanometer-thin poly(dimethylsiloxane) film. In this device, voltage-induced reconfigurations translate into a nonlinear, time-dependent electrical response. We use these temporal dynamics, arising from the viscoelastic memory of the polymer, to demonstrate an artificial neuron. As neural functionalities are embedded within the intrinsic material properties, energy efficiencies beyond those of biological systems are projected with much smaller active areas. Overall, our work establishes a design framework for extremely scaled mechanical tunability, opening emerging applications in energy-efficient, bioinspired computing, and intelligent materials and systems.

## INTRODUCTION

While conventional electronics with fixed architectures process information predominantly through the flow of electrons, compliant biological systems leverage physical transformations as part of their low energy, distributed, and adaptive information processing. Mechanical mechanisms have long played a role in the development of computing platforms, from the ancient Antikythera mechanism to Vannevar Bush’s differential analyzer (*1*). The gears and wheels of these early platforms have since been replaced by intricate mechanical transformations embedded within materials and structures, more closely resembling those of biological systems, enabling emerging avenues for unconventional computing (*2*–*11*). These mechanisms offer the potential for high energy efficiency, reconfigurability, robustness in harsh environments, and the capacity to embed nature-inspired intelligence within synthetic systems (*2*, *12*). Such features can help overcome key limitations of conventional electronic computing in meeting the growing demands of artificial intelligence, edge computing, and autonomous machines.

Mechanical information processing requires structures that are capable of geometric reconfiguration. External stimuli drive physical transitions in these structures, modulating mechanical, electrical, or optical responses. Such multimodal structure–function transformations can encode computing operations. For example, the discrete states of a multistable element or the resonance frequency response of a dynamically driven system can represent digital bits, enabling memory and logic (*5*, *6*, *13*–*16*). Such mechanisms have enabled active mechanical metamaterials that perform computation (*6*, *16*–*18*). When combined with responsive materials, these architectures further offer opportunities to integrate sensing, processing, and actuation, embodying a physical analog of biological intelligence (*2*, *19*, *20*). The nonlinearities inherent to engineered mechanical responses also support analog computation, aligning with emerging neuromorphic frameworks and their distributed information processing. Physical neural networks implemented in mechanical systems have been demonstrated, showing potential to outperform electronic counterparts (*3*, *8*, *21*).

These mechanical computing systems have been largely limited to millimeter scales. Miniaturizing to the nanoscale introduces richer and more finely tunable mechanical responses while enhancing integration potential and energy efficiency. Advances in semiconductor manufacturing have led to micro- and nanoelectromechanical relays and resonators for ultralow-power memory and logic (*22*–*28*). Microresonators have also been successfully used as physical reservoirs (*9*, *10*) and to realize continuous-time recurrent neural network dynamics for neuromorphic computing (*11*). However, the ultimate scaling to nanometer dimensions remains a fundamental challenge as surface adhesive forces compromise device structural stability and controlled tunability (*29*).

We address this challenge by leveraging nanoscale soft matter to enable mechanical reconfigurability at the subnanometer scale. These materials can support large mechanical deformations with programmable nonlinearities, providing a broad design space for in-material computation. We demonstrate this concept in the form of an electromechanically tunable tunneling junction composed of a compliant nanometer-thin poly(dimethylsiloxane) (PDMS) film. This device presents a time-dependent, nonlinear electrical response governed by the PDMS viscoelastic properties, which we use to realize an artificial nanomechanical neuron allowing energy-efficient bioinspired computing functions. This implementation of soft nanomechanical computing can be extended to other designs and transduction mechanisms, establishing a new platform for computing materials and systems.

## RESULTS

### Device design and electromechanical tuning

**Fig. 1.**
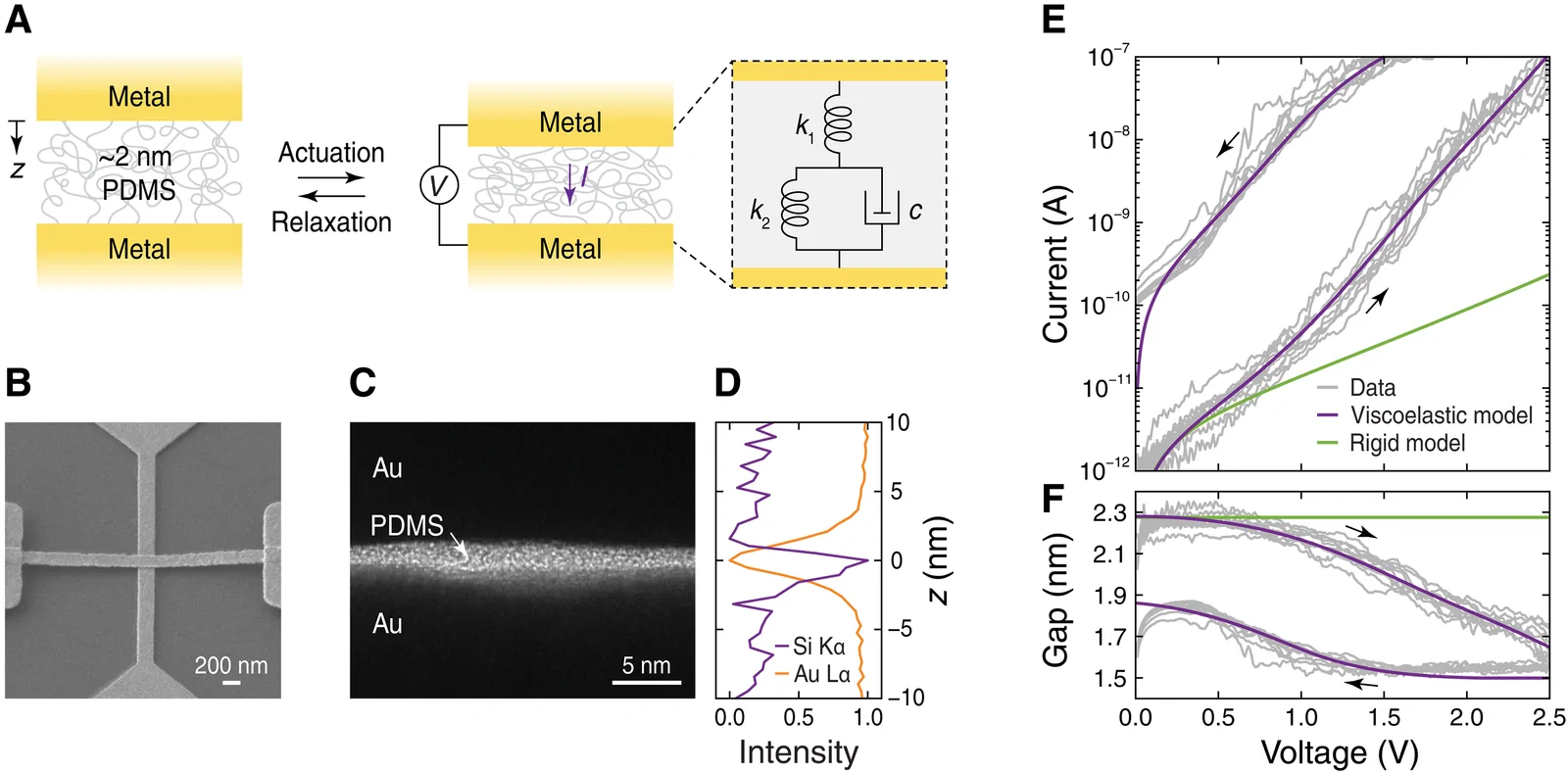
A viscoelastic nanoelectromechanical tunneling device. (**A**) Schematic of the device composed of a tunneling junction made from ∼2-nm-thin film of surface-grafted PDMS. An applied voltage-induced electrostatic force compresses the PDMS spacer, reducing the electrode spacing and increasing the tunneling current through the junction. As the applied force is removed, the device relaxes to its resting state assisted by the restoring force of the PDMS. The viscoelastic mechanical response of the PDMS is described by a lumped parameter model consisting of a spring that represents the instantaneous elastic response connected in series with a Kelvin element with a spring and a dashpot, representing the delayed viscoelastic response. $k_{1}$ and $k_{2}$ are the spring constants and c is the damping coefficient. (**B**) SEM of a representative device fabricated in a gold crossbar design with (200 nm)^2^ active region. (**C**) TEM cross section of the device showing gold electrodes separated by an ∼2-nm-thick PDMS spacer. (**D**) EDS line scan showing a decrease in Au Lα peak and appearance of Si Kα in the gap, confirming successful integration of the PDMS film. (**E**) Current-voltage relation (*I*-*V*) cycling of a characteristic device showing a nonlinear conduction and hysteresis. This behavior is inconsistent with a rigid tunneling junction (green line), however, is reproduced when considering mechanical modulation using the viscoelastic model (purple line) shown in (A). (**F**) Tunneling gap in the device during operation extracted from the *I*-*V* measurements using the Simmons tunneling model (text S2). The fits to the rigid and viscoelastic models are shown.

Our prototypical device (Fig. 1A) consists of an ∼2-nm-thick PDMS film connecting two gold electrodes. The top electrode is designed to be free to move such that the electrostatic force induced by an applied voltage across the electrodes causes compression of the PDMS spacer. Here, the presence of PDMS both enables the formation of a stable junction and controls its mechanical reconfiguration by offering a restoring force to balance the surface adhesive forces. This allows us to realize mechanically active devices with dimensions much smaller than conventional nanoelectromechanical systems. We designed the devices such that the performance is dominated by the mechanical properties of the polymer spacer, verified through the atomic force microscopy (AFM) force spectroscopy in fig. S1. To achieve films with nanometer-scale thickness, we deposited the PDMS on the electrode through a surface-grafted polymerization approach (fig. S2). The PDMS was further cross-linked to tune its mechanical response and enhance stability. The detailed fabrication procedure is described in Materials and Methods and in ref. (*30*). An example 200-nm-wide device with a crossbar geometry is shown in Fig. 1B. The active area consists of a PDMS junction ∼2 nm thick, as confirmed by the cross-sectional transmission electron microscope (TEM) image in Fig. 1C and the corresponding energy-dispersive x-ray spectroscopy (EDS) scan (Fig. 1D). This thickness is consistent with the 1.8-nm average thickness measured ellipsometrically.

In this design, compression of the PDMS reduces the electrode separation (g) leading to an increase in the tunneling current *I*, where $I\propto e^{-\beta g}$ with β representing the tunneling decay coefficient (*31*). This results in a nonlinear electrical response characterized by a large modulation in current and a hysteresis as shown in Fig. 1E. Similar performance is observed across independently processed samples (fig. S3), with variations that could be attributed to surface roughness or material inhomogeneities. The extent of nonlinearity can be tuned through the specific mechanical characteristics of the soft spacer (text S1 and figs. S4 and S5). Assuming a conduction dominated by direct tunneling, we use the Simmons model (*31*, *32*) to extract the tunneling gap at each applied voltage, as described in text S2. The results show a change in the gap from an initial 2.3-nm thickness to 1.6 nm (Fig. 1F), corroborating the expected electromechanical compression.

**Fig. 2.**
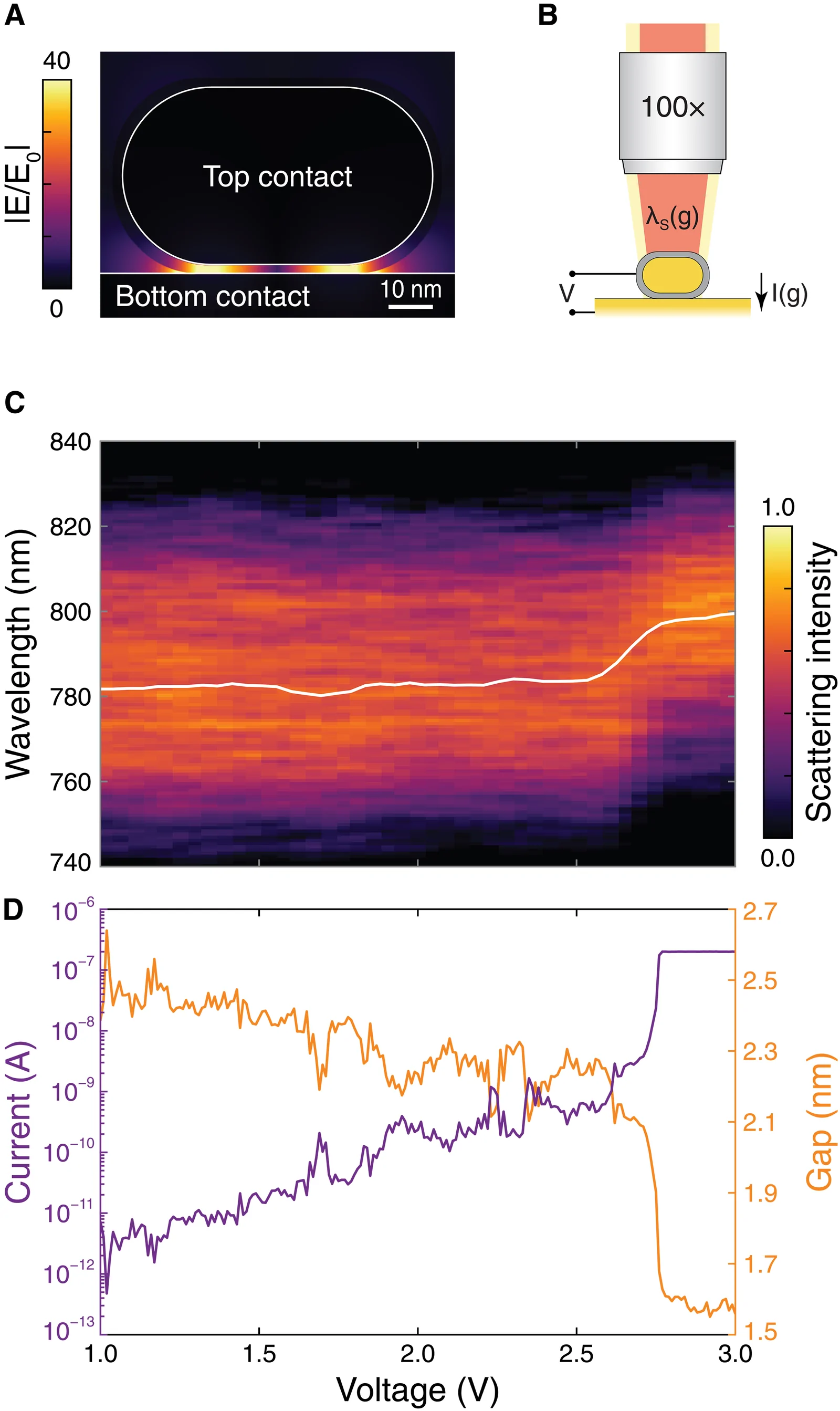
Optical validation of the working mechanism. (**A**) Simulated electric field enhancement of the device enabling a built-in platform to characterize mechanical changes in the gap using the plasmonic ruler feature of the structure. (**B**) Schematic of the metrology platform accommodating simultaneous electrical and dark-field scattering measurements. The voltage-induced decrease in the gap (g) is expected to increase the current and cause a red shift in the scattering resonance ($\lambda_{S}$). (**C**) Dark-field scattering as a function of applied voltage, showing 18-nm red shift in the scattering spectrum as voltage is swept from 1 to 3 V. (**D**) Corresponding current measured through the device with a set compliance of 200 nA, showing large increase concurrent with the spectral shift. The tunneling gap extracted from the measured current shows ∼0.9-nm shift which is consistent with the ∼0.8-nm shift extracted from the optical data by comparison to the simulated response in text S3.

To further validate the mechanical origin of our device performance, we implemented optical characterization concurrent with electrical testing. Our device design forms a metal-insulator-metal plasmonic nanocavity with a resonance highly sensitive to the spacer thickness (Fig. 2A). Thus, the spectral shift in the dark-field scattering response of the device can be tracked to probe the changes in the PDMS thickness during electrical operation (Fig. 2B) (*33*, *34*). To minimize background scattering, we conducted these experiments with devices consisting of an ∼70-nm-wide top electrode and a continuous bottom electrode. As a voltage is swept from 1 to 3 V, an 18-nm red shift in the dark-field scattering peak is observed, concurrent with the increase in tunneling current (Fig. 2, C and D). Finite-difference time-domain simulation of the plasmonic response of our device shows that this spectral shift corresponds to a PDMS compression of ∼0.8 nm, which is consistent with the ∼0.9-nm change extracted from the correlated electrical results (Fig. 2D, text S3, and fig. S6). We did not observe such a shift when testing a control device fabricated with a rigid Al_2_O_3_ spacer instead of the compliant PDMS (fig. S7).

### Proposed working mechanism

The mechanical modulation of the device is governed by the balance of nanoscale forces. The PDMS is compressed because of a combination of the applied electrostatic and intrinsic van der Waals forces (*35*, *36*)

$$F_{\text{act}}\left(z,V\right)=\frac{A_{H}A}{6\pi {\left(g_{0}-z\right)}^{3}}+\frac{{\kappa \varepsilon }_{0}A}{2{\left(g_{0}-z\right)}^{2}}V^{2}$$ (1)

where z is the extent of compression relative to the uncompressed electrode separation $g_{0}$, V is the applied voltage, A is the device area, $A_{H}$ is the Hamaker constant, and $\kappa \epsilon_{0}$ is the permittivity of the polymer spacer.

This actuating force ($F_{\text{act}}$) is counteracted by a mechanical restoring force ($F_{\text{rest}}$) provided by the polymer spacer. Thus, the mechanical response of this layer is critical in determining the device performance. Specifically, PDMS is viscoelastic, combining elastic and viscous properties, leading to a time-dependent response. This behavior can be captured by a lumped parameter model featuring a spring representing the instantaneous elastic response connected in series with a Kelvin element, consisting of a spring and a dashpot, representing the delayed viscoelastic response (Fig. 1A). This collective behavior is described by (*37*)

$$F_{\text{act}}\left(z,V\right)=F_{e,1}\left(z_{1}\right)=F_{e,2}\left(z_{2}\right)+c\frac{dz_{2}}{\mathrm{dt}}$$ (2)

where $F_{e,1}$ and $F_{e,2}$ are the elastic restoring forces provided by the two springs and the last term describes the viscous damping force with coefficient c. To account for the compression stiffening (*38*), our model uses nonlinear springs to define $F_{e,1}$ and $F_{e,2}$

Fe1,2(z1,2)={k1,2z1,2, z1,2≪gc1,2∞, z1,2→gc1,2 (3)

such that the response is linear at small strains with spring constants $k_{1}$ and $k_{2}$, and full stiffening occurs as a finite compressible gap ($g_{c1,2}$) is reached. This model sets a characteristic timescale, $\tau =c/k_{2}$, where viscoelastic effects are pronounced. A detailed discussion of the model is included in text S1.

The nonlinear electrical conduction and the hysteresis observed in our devices are well described by this model, as indicated by the best fit to the experiment (Fig. 1E). Here, our device performance is consistent with a PDMS spacer with a viscoelastic time constant of 4.9 s, and an instantaneous and a delayed modulus of 23 and 6.2 MPa, respectively, derived from the fitted $k_{1}$ and $k_{2}$ as described in text S1. All fitted parameters are summarized in table S1. The mechanical properties extracted from the fit to electrical measurements are in line with the 10-MPa modulus experimentally measured using AFM force spectroscopy (fig. S1 and text S1).

**Fig. 3.**
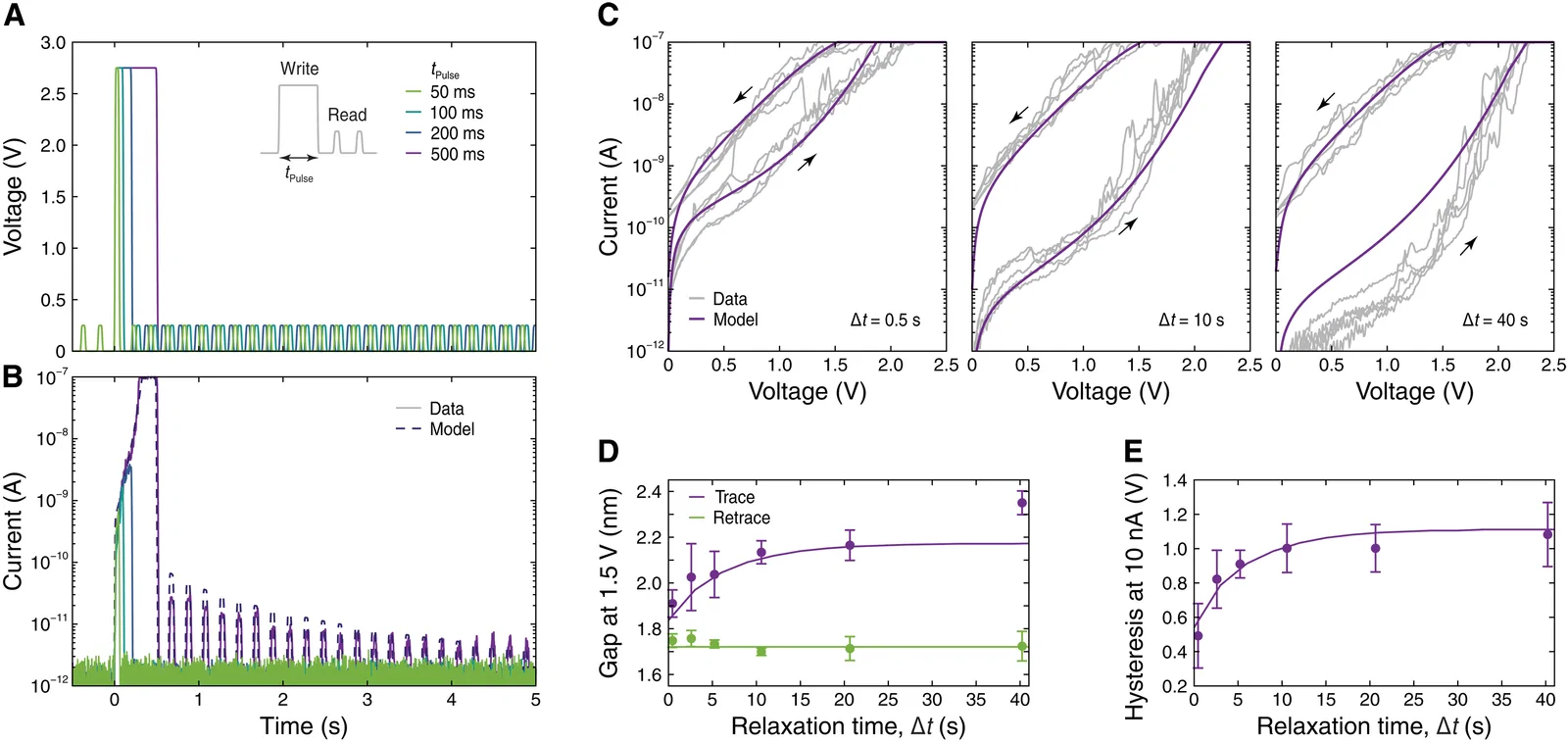
Viscoelastic device performance. (**A**) Applied voltage sequence where 2.75-V write pulse is applied for 50 to 500 ms, followed by series of 0.25-V read pulses. (**B**) Corresponding current through the device, where substantial displacement only occurs once pulse approaches viscoelastic timescale. Viscoelastic model reproduces the observed behavior with time constant of 4.5 s. (**C**) *I*-*V* characterization of device with wait time between sequential measurements (∆t) varied from 0.5 to 40 s, showing increased starting current and hysteresis as the relaxation time gets longer. (**D**) Modeled and extracted tunneling gap at 1.5 V for the device in (C) as a function of relaxation time, in trace and retrace segments. Initial gap is decreased at shorter relaxation times due to viscoelastic memory. (**E**) Modeled and extracted hysteresis at 10 nA as a function of relaxation time. Here, hysteresis is defined as the voltage difference between the first points where the data cross 10 nA in the trace and retrace segments. Error bars in (D) and (E) represent standard deviation of five sequential measurements.

The viscoelastic properties of PDMS provide a nonlinear, time-dependent mechanical reconfiguration that defines the device performance. This effect can be directly probed through measuring the change in current over time in response to an applied actuation voltage (Fig. 3, A and B). A short voltage pulse, 2.75 V for 50 ms, minimally compresses the PDMS by ∼8%, corresponding to a small change in current with spontaneous recovery upon voltage removal. As the pulse duration increases and approaches the viscoelastic timescale, PDMS compresses more. The exponential gap dependence of conductance leads to an increase in the measured current. For example, application of a 500-ms pulse results in ∼30% compression because of both the instantaneous elastic and viscoelastic responses, followed by a viscoelastic relaxation that is characterized by a gradual recovery of the current to its original state. Fitting this data yields a viscoelastic relaxation time of 4.5 s.

This temporal response introduces memory in the device such that the performance is not only dependent on the instantaneous applied voltage but also the history of previous actuations. This is evident through the change in the electrical response as the wait time between sequential current-voltage relation (*I*-*V*) measurements is adjusted (Fig. 3C). With reduced wait time from 40 to 0.5 s, incomplete relaxation results in the gradual decrease in the starting gap, as evidenced by an increase in the current (Fig. 3D). Combined with polymer stiffening, this leads to a reduced hysteresis in subsequent runs (Fig. 3E). This effective device memory can be used to realize bioinspired computing functions.

### In-material neuromorphic computing

**Fig. 4.**
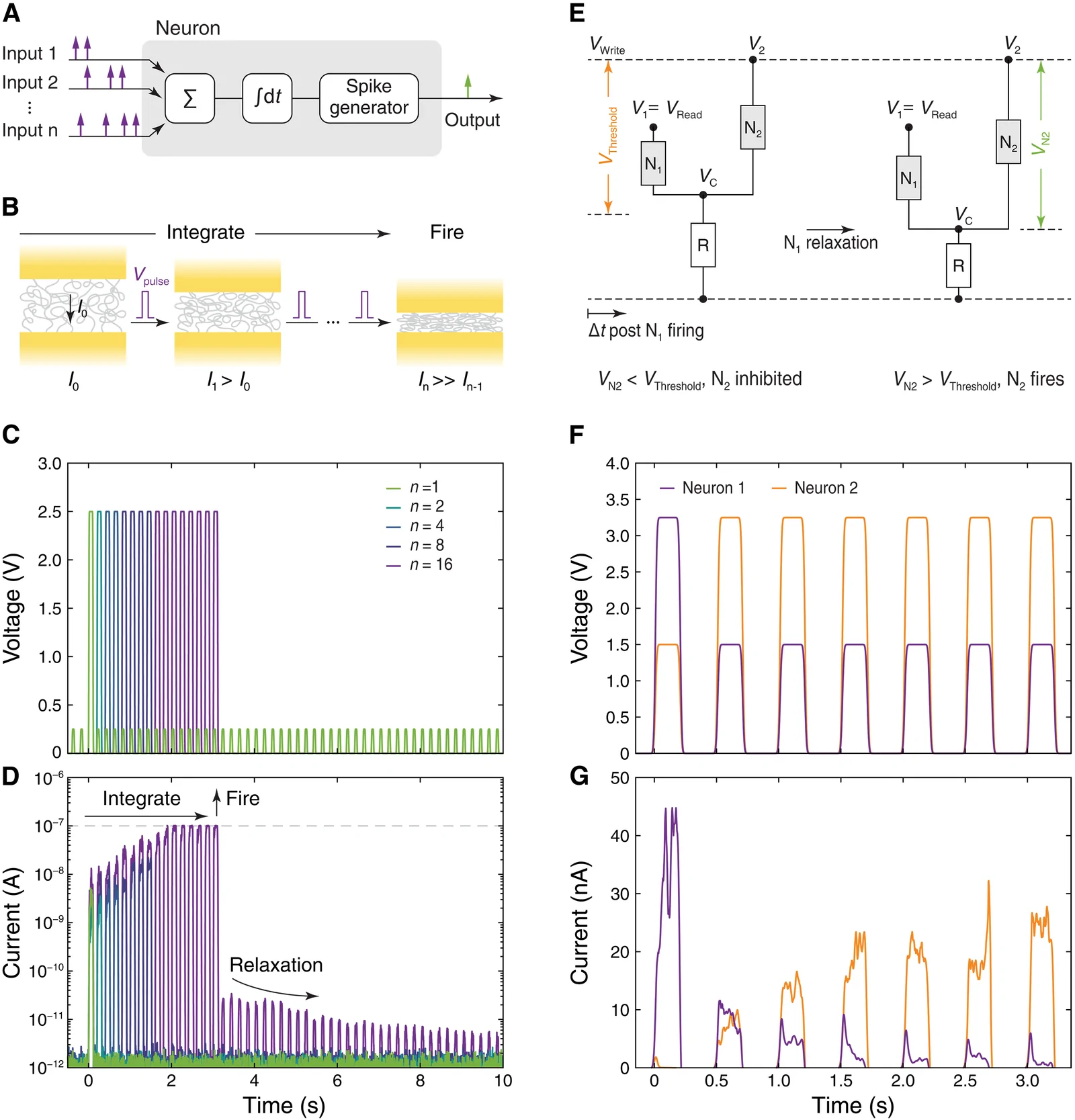
In-material neuromorphic computing. (**A**) Schematic illustration of leaky integrate-and-fire (LIF) neuron behavior, where neuron spatially and temporally integrates inputs, and fires when stimuli cross a set threshold. (**B**) Schematic illustration of nanomechanical integrate and fire, where sequential subthreshold pulses applied below the viscoelastic timescale compress the device, potentiating it and eventually causing it to fire. (**C**) Voltage pulse sequence used to demonstrate LIF neuron, where 1 to 16 2.5-V pulses are applied to potentiate the device, followed by 0.25-V read pulses. (**D**) Corresponding read current, where the device fires only after 16 pulses are applied. The dashed line shows the compliance current of the measurement setup. (**E**) Schematic illustration of inhibitory coupling, where nanomechanical neurons 1 (N_1_) and 2 (N_2_) are connected to a shared load resistor (R) at node $V_{C}$. When neuron 1 fires, the voltage-drop across neuron 2 ($V_{N2}$) is insufficient to induce firing; hence, neuron 2 is inhibited. As neuron 1 relaxes, $V_{N2}$ increases, and once it exceeds the threshold $V_{\text{Threshold}}$, neuron 2 can fire. (**F**) Voltage pulses applied to neurons 1 and 2, to first write to neuron 1 and then attempt to write to neuron 2. In this example, $V_{\text{Write}}$ = 3.25 V, and $V_{\text{Read}}$ = 1.5 V. (**G**) Current measured through neurons 1 and 2, showing inhibition of writing to neuron 2 immediately after neuron 1 fires.

Neurons play an essential role in energy-efficient information processing in biological systems by receiving, integrating, and transmitting information through spiking action potentials (*39*, *40*). A neuron temporally integrates input spikes received through the synapses. Because of membrane potential leakage, the integrated signal decays toward its resting state in the absence of sufficient stimulation. However, when input signals arrive at sufficiently short intervals, the membrane potential builds up, and once a threshold is reached, an action potential (or spike) triggers and propagates down to other neurons. Following this firing event, the neuron resets to its resting state. Emulating this leaky integrate-and-fire behavior, schematically outlined in Fig. 4A, is central to neuromorphic computing (*40*). The temporal dynamics of our device enables efficient replication of this neuronal function through its intrinsic viscoelasticity driven by the polymer spacer (Fig. 4B), where the compression depends on both the magnitude and duration of the applied electrostatic force.

Figure 4 (C and D) shows the device response to a sequence of subthreshold voltage pulses. If applied with a frequency faster than the relaxation time constant of the PDMS, the voltage pulses result in a continuous decrease in the electrode spacing and a corresponding increase in the current. Here, 16 pulses build up to a sufficient integrated force to cause the device to fire. The firing event is followed by the device gradually relaxing back to its resting state at the end of the pulse sequence. The relaxation dynamics and integration timescale are set by the viscoelastic properties of the polymer spacer, corresponding to a viscoelastic memory in the neuron. This memory, which is on the order of seconds, is longer than most common artificial neuron demonstrations in the micro- and milliseconds range (table S2), making the platform suited for tasks requiring temporal context in this regime (*41*). This timescale is further tunable across a broad range through chemical engineering of viscoelasticity (*42*).

In our viscoelastic nanomechanical artificial neuron, the key features of leaky integration and threshold firing are encoded within a single device through its intrinsic materials properties. Thus, the need for auxiliary components, including capacitors and complex circuitry that have commonly been necessary for similar demonstrations of neuronal functions (table S2), is eliminated. This enables improvements in scalability, integration density, and energy efficiency prospects. We measure an energy consumption of 54 nJ in a device with an active area of 200 nm by 200 nm, enabling high integration densities. Fundamentally, our demonstrated devices require ∼2 fJ of electrical and ∼0.1 fJ of mechanical energy to be stored per firing event, as discussed in text S4. Thus, with reductions in leakage and further scaling, this approach can surpass the energy efficiency of biological neurons in a device with a <0.04-μm^2^ active area, >100 × smaller than a typical neuron (∼5 to 100 μm in diameter) (*43*–*45*). The smaller footprint and potential for energy efficient performance while offering longer embedded memory also stand out when compared to spiking neuron technologies using other material systems and working mechanisms (table S2).

Next, we demonstrate inhibitory coupling between two devices, exemplifying the neuronal interactions that are needed for neuromorphic computing. We implement this coupling to achieve lateral inhibition, a fundamental mechanism in which excitation of one neuron suppresses the activity of its neighbor (*46*). We achieve this by connecting two parallel devices at a common node ($V_{C}$) to a load resistor as shown in Fig. 4E. The value of the resistor is selected to be larger than the postfiring resistance of the device to allow the firing of one device to modulate the other (*47*, *48*). Such inhibitory action helps regulate and stabilize neuronal activities, improve input discrimination, and enable competitive processing (*46*, *49*).

As shown in Fig. 4 (E to G), to demonstrate the inhibitory performance, we excite neuron 1 through a voltage pulse with voltage $V_{\text{Write}}$ applied to node $V_{1}$ while probing its state using a series of read pulses with voltage $V_{\text{Read}}$. After writing to neuron 1, a series of write pulses are sent to node $V_{2}$ to attempt to write to neuron 2. The write and read voltages are selected such that $V_{\text{Write}}$ causes the neuron to fire, while $V_{\text{Write}}-V_{\text{Read}}$ remains below the firing threshold. Once neuron 1 fires, its resistance drops far below R, pulling $V_{C}$ to $V_{1}$. This results in the voltage drop across neuron 2 ($V_{N2}$) becoming insufficient to reach the firing threshold. $V_{\text{Read}}$ is further chosen such that the change in the device conduction is sufficient to cause a substantial drop across the load resistor. Thus, neuron 2 is inhibited from firing after neuron 1 has fired for a duration determined by the viscoelastic memory of neuron 1 arising from the polymer spacer mechanical properties. Such dynamic inhibitory action can be leveraged to enable temporal classification tasks.

## DISCUSSION

Dynamic control of nanoscale mechanics introduces a highly sensitive and versatile mechanism for tuning the nonlinear properties of materials and structures, enabling new approaches to information processing. Although micro- and macroscale mechanical systems have shown promise, scaling to nanometer dimensions—where ultimate sensitivity and efficiency can be realized—has been fundamentally hindered by a lack of structural stability and control. By leveraging nanoscale soft matter, we overcome this challenge and achieve subnanometer structural control and tunability. Notably, in contrast to the rigid materials commonly used in nanoelectromechanical systems, soft matter provides a broad design space for engineering mechanical performance through chemical and thin-film approaches.

Using the viscoelastic memory in a polymer spacer, we demonstrate a device with nonlinear conduction characteristics and temporal dynamics that realizes neuromorphic functionalities embedded within intrinsic material properties. As the need for peripheral electrical components and complex circuitry are reduced, compact footprints with high energy efficiency can be realized. Our platform is projected to enable ∼2 fJ per firing event, surpassing the energy efficiency of a biological neuron with a much smaller footprint. Practical realization of these prospects will advance through simultaneous developments in engineering of the soft matter, device and system design, and training strategies.

The chemical versatility of soft matter further enables material-level cointegration of functionalities such as sensing and computing to support more complex intelligent tasks (*20*). In addition, mechanical systems are conducive to transduction mechanisms beyond the electrical domain, including thermal (*33*), chemical (*50*), and optical (*51*) modalities, thereby expanding the application space. Overall, our platform introduces an approach to engineering nanomechanical systems at extreme levels of scaling with diverse degrees of mechanical control, opening opportunities for energy-efficient bioinspired computing materials and systems for intelligent robots, autonomous machines, and edge computing. The structural and chemical biocompatibility of soft matter further makes our platform well suited for interfacing with biological systems, helping to bridge the increasingly important gap between artificial and natural systems.

## MATERIALS AND METHODS

### Device fabrication

A soft contact approach was used to fabricate the devices (*30*). First, the device scaffold consisting of top and bottom electrodes separated by a gap was fabricated; then, upon growth of the PDMS film, the top electrode was collapsed to form the desired metal-PDMS-metal structure.

The top and bottom electrodes were fabricated on a SiO_2_ substrate using electron-beam lithography followed by metal lift-off. The patterning was conducted on an Elionix HS50 using poly(methyl methacrylate) electron-beam resist (950K A4, Kayaku), with a layer of DisCharge H_2_O 2X (DisChem). The patterned resist was developed in 3:1 isopropanol:methyl isobutyl ketone (MIBK, Transene). The surface was then treated by oxygen reactive ion etching in a SAMCO RIE-230iP to remove resist residues from the developed areas. This was followed by electron-beam deposition of metal layers (Temescal, FC-2000) and their lift-off in *n*-methyl-2-pyrrolidone (PureSolv). The bottom electrodes consisted of 2 nm/40 nm Ti/Au, and the top electrodes consisted of 40 nm Au. The wires and pads for the top electrodes were formed from 1 nm/60 nm Cr/Au. Upon fabrication of the bottom electrodes, they were sequentially coated in 5 nm of atomic layer deposited Al_2_O_3_ (Veeco Savannah ALD) to protect the electrode surface, 3 nm of evaporated Cr as an etch stop, and 100 nm of spin-coated hydrogen silsesquioxane (HSQ, 6% in MIBK, Dow Corning) as the gap spacer. The HSQ was cured at 240°C for 10 min before fabricating the top electrodes, wires, and pads.

To form the devices, the sacrificial HSQ spacer layer was undercut in diluted buffered oxide etchant (BOE, Fujifilm), and then the exposed electrode surface was cleaned in 3:1 piranha solution. Subsequently, the Cr etch-stop layer was removed in chromium etchant 1020 (Transene). The PDMS film was then grown as described in the following section. Last, the protective Al_2_O_3_ layer was etched in BOE, and the sample was rinsed in deionized water. The sacrificial Al_2_O_3_ layer limited the PDMS grafting to a single electrode surface. Once rinsed, the device was removed from water and dried under nitrogen. During this drying process, capillary forces collapse the top electrode into soft contact with the bottom electrode, forming the desired metal-PDMS-metal structure. Scanning electron microscopy (SEM) images of a suspended device and a device after contact formation are presented in fig. S8. The device is continuously kept wet between initial BOE undercutting and final drying to avoid premature capillary collapse. This soft contact approach avoids damage to the PDMS spacer during device formation while ensuring that the top electrode is free to move. In this design, the top beam has a spring constant of <3 nN/nm, as measured through AFM force spectroscopy, substantially less than the ∼50 nN/nm spring constant of the PDMS junction (fig. S1).

### PDMS film deposition

Nanometer-thin PDMS films were prepared through surface-initiated polymerization from a (3-mercaptopropyl)trimethoxysilane (MPTMS) self-assembled layer. The process is summarized in fig. S2. Unless otherwise specified, all reagents were purchased from MilliporeSigma. The MPTMS layer was assembled on the device surface by submersing it in a 0.1 mM ethanolic solution of MPTMS for 30 min in a nitrogen glovebox. Subsequently, the samples were submersed in clean ethanol and removed from the glovebox before being transferred to water. They were then incubated for 15 min in 10 mM aqueous hydrochloric acid to hydrolyze the methoxysilanes, forming reactive silanol groups (*52*). Samples were transferred from water to ethanol to toluene. PDMS was polymerized from the terminal silanol groups based on a modified procedure from ref. (*53*). Briefly, wet toluene was prepared by bubbling nitrogen saturated with water vapor through toluene overnight and used to make a 200 mM solution of 1,3-dichlorotetramethyldisiloxane (Thermo Fisher Scientific). Samples were immersed in this solution for a 60-min polymerization. Upon growth, the samples were submersed in clean toluene and mildly agitated to rinse the surface.

Cross-linking was performed using benzoyl peroxide (BPO, Luperox A98) as a radical initiator (*54*). Samples were submersed in 20 mM BPO in anhydrous toluene (Extra Dry, AcroSeal, Thermo Fisher Scientific), which was then sparged with nitrogen for at least 1 hour before cross-linking. Cross-linking was performed in a 110°C oil bath on a hotplate for 2 hours. Samples were then rinsed sequentially in toluene, ethanol, and deionized water.

### Rigid control for optical experiment

We fabricated a device with a rigid Al_2_O_3_ spacer in place of the mechanically compliant PDMS film. Devices were fabricated as described above but without including the Cr etch stop. Following BOE undercutting of the HSQ layer and cleaning of gold electrode surfaces in piranha solution, the sample was critical-point dried (Tousimis Autosamdri 931). A 0.6-nm Al_2_O_3_ film was grown on the electrode surfaces using atomic layer deposition (Veeco Savannah ALD) forming a gap ∼1.2 nm thick upon capillary collapse.

### X-ray photoelectron spectroscopy

X-ray photoelectron spectroscopy spectra were acquired using a Thermo Fisher Scientific K-Alpha+ system with an AlKα monochromated source (hυ = 1486.6 eV). Samples were attached to the stage using copper contacts. Measurements were taken at a 400-μm spot size with a 12-kV anode potential and 6-mA emission current with charge neutralization. The base pressure during measurement was <8 × 10^−8^ mbar, and the surface normal-to-detector angle was 90°. High-resolution spectra were acquired using a 50-eV pass energy and a 0.1-eV step size. The dwell time at each point was 25 ms. To improve the signal-to-noise ratio, the high-resolution Si 2p spectra were averaged over 10 consecutive measurements, and the S 2p spectra were averaged over 20 consecutive measurements. Binding energies were referenced to the Au4f_7/2_ peak at 84.0 eV.

### Ellipsometry

The thickness of the PDMS film grafted on the gold surface was characterized using a J. A. Woollam M-2000 spectroscopic ellipsometer. A gold baseline was first measured and fitted using a B-spline. The PDMS thin films were then measured and fitted assuming a constant refractive index of 1.45, using the gold model as the substrate. Measurements were performed at 65°, 70°, and 75° angles of incidence.

### AFM imaging and force spectroscopy

Samples to characterize the surface roughness were prepared with the exact conditions used in device fabrication. AFM imaging and force spectroscopy was conducted on an Asylum Cypher S system. AFM imaging was conducted with 160 AC-NA tips (OPUS). Force spectroscopy experiments were conducted with 240 AC-NA tips (OPUS). The tip spring constant and inverse optical lever sensitivity were thermally calibrated. To assess the mechanical properties of our device scaffold, the tip was pressed against a suspended beam 10 times, and the force-indentation curves were recorded. To assess the compressibility of our film, 10 sequential force-indentation curves were recorded with the same tip pressing either against the overlap region of the device or against a bare Au electrode. The additional indentation in the overlap region represents the mechanical response of the polymer film, allowing estimation of the effective elastic modulus of the film: $E\approx Fg_{0}/A\delta$, where F is the measured force, $g_{0}$ is the thickness of the PDMS film, A is the overlap area of the junction, and δ is the indentation.

### Electron microscopy

SEM imaging was performed with a Zeiss Gemini 450 SEM at 2-kV accelerating voltage, 100-pA current, and the secondary electron detector. Devices for TEM imaging containing a PDMS film were fabricated using the approach described above. Focused ion-beam cross-sectioning of a device with a 200-nm-wide top electrode was performed using an FEI Helios 660 (Thermo Fisher Scientific) system. First, protective layers of carbon (∼50 nm) and platinum (∼500 nm) were electron-beam deposited on the device, followed by an additional ∼1 μm of platinum ion-beam deposited. Cross-sectioning was performed using Ga ions, resulting in a <100-nm-thick lamella for TEM imaging. The lamella was imaged with a JEOL ARM 200F scanning TEM at an accelerating voltage of 200 kV. EDS mapping was performed using an EDAX Octane detector with an integration time of 15 min.

### Electrical measurements

All electrical measurements were conducted using a Keithley 2636B source measurement unit, with custom control code. *I*-*V* cycling was conducted at a rate of 2 V/s. Measurements were conducted in a Nextron micro probe system (PZMPS-PT4C). All electrical measurements were from devices with an area of 200 nm by 200 nm and were measured under nitrogen, with a relative humidity of 80%, and with a 100-nA compliance. We found that devices showed enhanced stability in humid environments compared to dry environments, which we attribute to the water vapor solvating the polymer film (*55*). Here, the inhibitory coupling demonstration was implemented with a discrete 50-MΩ resistor; however, this same coupling can be achieved through on-chip fabricated polymeric, molecular, or inorganic resistors.

### In situ dark-field scattering spectroscopy

Dark-field scattering spectroscopy was conducted on a WITec Alpha300 confocal spectroscopy system. The measured spectra were normalized to the spectra of a Spectralon reflectance standard (Labsphere) to correct for the instrument’s varying collection efficiency over wavelength (*56*). The excitation and collection pathways were polarized normal to the long axis of the top electrode. Measurements were conducted under humid nitrogen. The measured device had an ∼70-nm-wide top electrode, and a uniform bottom electrode, with an overlap area of approximately 70 nm by 1 μm. The device was excited electrically using a Keithley 2636B source measurement unit, with a 200-nA compliance. The peak position shown as a white line in Fig. 2C was extracted from the maximum of a polynomial fit to the measured data.

## Supplementary Materials

### This PDF file includes:

Supplementary Text S1 to S4 Figs. S1 to S8 Tables S1 and S2 References
