# Supplementary material for: Viscoelastic nanomechanical devices for neuromorphic information processing
Source: Sci Adv. 2026 Sep 16;12(38):eaeg9893. doi: 10.1126/sciadv.aeg9893 (PMC13580571; doi:10.1126/sciadv.aeg9893)
Supplement: Supplementary file 1 [file sciadv.aeg9893_sm.pdf]

Science Advances
AAAS

Supplementary Materials for

Viscoelastic nanomechanical devices for neuromorphic information processing

Peter F. Satterthwaite *et al.*

Corresponding author: Farnaz Niroui, fniroui@mit.edu

Sci. Adv. **12**, eaeg9893 (2026)
DOI: 10.1126/sciadv.aeg9893

This PDF file includes:

Supplementary Text S1 to S4
Figs. S1 to S8
Tables S1 and S2
References

Supplementary Text

Supplementary Text S1. Electromechanical modeling of the device

S1.1 – Force balance describing the device response

The force actuating our devices stems from both the intrinsic van der Waals forces and the applied electrostatic force (*35*, *36*):

$$F_{\mathrm{act}}(z,V) = \frac{A_{\mathrm{H}}A}{6\pi(g_0 - z)^3} + \frac{\varepsilon_0 \kappa A}{2(g_0 - z)^2}V^2 \tag{S1}$$

where z is the extent of compression relative to the uncompressed electrode separation g_0, V is the applied voltage, A is the device area, A_{H} is the Hammaker constant and $\kappa\epsilon_0$ is the dielectric constant of the polymer spacer. For our modeling, A_{H} was set to 3.12×10^{-19} J based on the literature value for gold interacting across an organic layer (*57*), and κ was set to 2.2 as reported for PDMS (*58*).

This actuating force is balanced by the viscoelastic restoring force provided by the polymer spacer. We modeled the polymer's viscoelastic behavior using a lumped mechanical representation comprising a spring that describes the instantaneous elastic response connected in series with a Kelvin element, consisting of a spring and a dashpot, describing the delayed viscoelastic response (Fig. 1A). This collective behavior is given by (*37*):

$$F_{\mathrm{act}}(z,V) = F_{\mathrm{e},1}(z_1) = F_{\mathrm{e},2}(z_2) + c\frac{dz_2}{dt} \tag{S2}$$

where $F_{\mathrm{e},1}$ and $F_{\mathrm{e},2}$ are the elastic restoring forces provided by the two springs and the last term describes the viscous damping force with coefficient c. In the simplest case of using linear springs, $F_{\mathrm{e},1} = k_1 z_1$ and $F_{\mathrm{e},2} = k_2 z_2$ where k_1 and k_2 are the spring constants and z_1 and z_2 are the displacements corresponding to each of the springs. However, the PDMS film in our device has a finite compressible gap (g_{c}) arising from factors including the excluded volume of the polymer chains and the rigidity of the anchor groups used for their surface grafting (*59*). As a result, a stiffening is expected during actuation which our model accounts for by implementing nonlinear springs. Many models exist to describe the nonlinear elasticity of polymers, though most are focused on bulk systems, with limited applicability to nanometer-thin films (*38*, *60*, *61*). We model our device with a restoring force, $F_{\mathrm{e}1,2}(z_{1,2}) = k_{1,2}g_{\mathrm{c}1,\mathrm{c}2}\tanh^{-1}(z_{1,2}/g_{\mathrm{c}1,\mathrm{c}2})$, where $g_{\mathrm{c}1/\mathrm{c}2}$ is the portion of the gap that is compressible under elastic or viscoelastic deformation, selected such that $g_c = g_{\mathrm{c}1} + g_{\mathrm{c}2}$. This relation is selected as it represents the desired asymptotic behavior:

$$F_{\mathrm{e}1,2}(z_{1,2}) = \begin{cases} k_{1,2}z_{1,2}, & z_{1,2} \ll g_{\mathrm{c}1,2} \\ \infty, & z_{1,2} \to g_{\mathrm{c}1,2} \end{cases} \tag{S3}$$

such that the response is linear at small strains, and full stiffening occurs as the finite compressible gap ($g_{c1,2}$) is reached. This model sets a characteristic timescale, $\tau = c/k_2$, where viscoelastic effects are substantial.

Combining all the relevant effects (electrostatic forces, van der Waals forces, viscoelasticity, finite compressibility), the devices were modeled with the following equations.

$$F_{\mathrm{act}}(z, V) = k_1 g_{\mathrm{c1}} \tanh^{-1}\left(\frac{z_1}{g_{\mathrm{c1}}}\right) \tag{S4}$$

$$F_{\mathrm{act}}(z, V) = k_2 g_{\mathrm{c2}} \tanh^{-1}\left(\frac{z_2}{g_{\mathrm{c2}}}\right) + c\frac{dz_2}{dt} \tag{S5}$$

We simulated the device performance in MATLAB by numerically evaluating Equations S4-S5 under the experimental applied voltage. This allowed us to simulate the PDMS spacer's vertical thickness over time, which we used to predict the tunneling current over time using the Simmons tunneling model, described in supplementary text S2. The device parameters ($k_{1,2}, \tau, g_0, g_{\mathrm{c1,c2}}$) were fitted using least-squares minimization of the logarithm of the modeled current compared to the logarithm of the measured current. Once fitted, the effective moduli ($E_{1,2}$) were evaluated using the modeled spring constants by $k_{1,2} = AE_{1,2}/g_{\mathrm{c}}$ (*37*). For any experiment (electrical characterization, AFM force spectroscopy) that is conducted at a finite rate, the effective spring constant (modulus) lies somewhere between k_1 (E_1), the spring constant (modulus) observed for infinitely fast excitation, and $k_1k_2/(k_1 + k_2)$ ($E_1E_2/(E_1 + E_2)$), the spring constant (modulus) observed for infinitely slow excitation. Thus, based on the parameters extracted from the device in Fig. 1, we expect AFM force spectroscopy to show a modulus between 4.9 MPa and 23 MPa, consistent with our measured 10 MPa modulus.

S1.2 – Nanomechanical viscoelastic memory

Our device dynamics, as described in Equations S1, S4 and S5, lead to a complex nonlinear temporal behavior. In particular, the hysteresis in the *I–V* response of our devices exemplifies a nanomechanical memory. This hysteresis arises from the combined effects of force-balance bistability and the viscoelastic properties of the soft polymer spacer. These two effects lead to a sweep frequency dependent hysteresis. The simulated *I–V* characteristics of devices as a function of sweep frequency is shown in fig. S4. At low frequencies relative to the viscoelastic time constant, devices can show hysteresis due to the force balance instability present in the electrostatic actuators, resulting in pull-in (*35*). Assuming a linear spring and no van der Waals forces/viscoelasticity, this hysteresis is analytically given by Equation S6.

$$V_{\mathrm{H}}|_{f\to 0} = \sqrt{\frac{2kg_{\mathrm{c}}}{A\epsilon_0\kappa}}\left(\sqrt{\frac{4}{27}\frac{g_0^3}{g_{\mathrm{c}}}} - (g_0 - g_{\mathrm{c}})\right) \tag{S6}$$

For this linear model, hysteresis is present as long as $g_{\mathrm{c}} > g_0/3$. The presence of van der Waals forces increases the hysteresis above this analytical value, while nonlinearities that stiffen the

polymer at large displacements decrease its magnitude. Viscoelasticity enhances hysteresis, which reaches a maximum when the sweep frequency is comparable to the viscoelastic time constant. At higher frequencies, the viscoelastic component becomes negligible, and the hysteresis diminishes (fig. S4).

The device performance can be engineered through the mechanical response of the polymer spacer. Two parameters are particularly important. First, the elastic modulus of the polymer can be adjusted: decreasing the modulus lowers the voltage required to actuate the device and increases hysteresis (fig. S5A). Second, for a given modulus, the compressible portion of the gap can be varied to control the hysteresis. As shown in fig. S5B, a larger compressible gap results in an increased hysteresis.

Supplementary Text S2. Simmons modeling of tunneling gap change

Our device, which consists of a ~ 2 nm-thin insulating PDMS spacer separating two electrodes, forms a quantum tunneling junction. The current through this junction can be modeled using the Simmons tunneling model, which has been shown to describe non-resonant tunneling through thin organic layers (*31*, *32*, *62*):

$$I = \left(\frac{qA}{4\pi^2\hbar g^2}\right)\left[\left(\phi - \frac{qV}{2}\right)\exp\left(-\frac{2g\alpha\sqrt{2m_{\mathrm{e}}(\phi - qV/2)}}{\hbar}\right) - \left(\phi + \frac{qV}{2}\right)\exp\left(-\frac{2g\alpha\sqrt{2m_{\mathrm{e}}(\phi + qV/2)}}{\hbar}\right)\right] \quad \text{(S7)}$$

where A is the device area, m_{e} and q are the mass and charge of an electron, $\hbar$ is the reduced Plank's constant, ϕ is the tunneling barrier, and α is a non-ideality factor. For our modeling, we set ϕ and α to 3.5 eV and 0.57, respectively, consistent with the reported literature values for tunneling through insulating organic layers (*63*). Using these values and the experimentally measured current, we applied Equation S7 to extract the tunneling gap at each applied voltage. The resulting gap change is presented in Fig. 1F, Fig. 2D and Fig. 3D.

Supplementary Text S3. Plasmonic nanocavity design, modeling and characterization

The plasmonic response of our structures was simulated using Ansys Lumerical finite-difference time-domain (FDTD) software. A two-dimensional slice of the device, consisting of a 70 nm-wide top gold electrode on a uniform bottom electrode was used for these simulations, with the spacing between the electrodes varied. The beam edges were rounded with a radius of curvature of 20 nm, consistent with the TEM analysis of the fabricated devices. The dielectric properties of gold were drawn from Johnson and Christy's data (*64*). The PDMS layer was modeled with a refractive index of 1.42 which corresponds to the refractive index of PDMS in the near IR (*65*). The structure was excited with plane waves at normal and parallel incidence, with the electric field polarized in-plane, and the scattering was recorded through a monitor with a numerical aperture of 0.75. To simulate our dark-field scattering experiments, a weighted sum of the two responses was used to model the light incident at 60º. The scattering as a function of the gap is shown in fig. S6A. As the gap decreases, the main scattering peak red shifts (fig. S6B), and narrows. This is consistent with

the experimental results, where the scattering at 3 V is red shifted and narrowed relative to the scattering at 1 V. The simulated spectra that best match the experimental spectra at 1 V and 3 V are those at 2.1 nm and 1.3 nm, respectively. This ~ 0.8 nm decrease in gap is consistent with the ~ 0.9 nm compression inferred from modeling the electrical data (Fig. 2D).

To further confirm the role of mechanics in the shift in dark-field scattering, we performed a control experiment where a voltage was applied across a device with Al_2O_3 tunnel junction, which is not mechanically active (fig. S7). The fabrication of this device is described in the Methods section. As expected, the tunneling current increases, but no shift in the dark-field scattering is observed, in contrast to our mechanically active devices.

<u>Supplementary Text S4. Energy consumption of the viscoelastic nanomechanical neuron</u>

The energy consumed to potentiate the device is given by $U = \int_0^{t_{\text{fire}}} I(t) \cdot V(t) dt$, where t_{fire} is the time needed to fire, and $I(t)$ and $V(t)$ are the current and voltage through the measured device. Here, t_{fire} is defined as the time needed to reach the compliance current (100 nA for the device shown in Fig. 4C-D). In this demonstration, the consumed energy is dominated by the leakage current. The fundamental limit to firing energy is set by the electrical and mechanical energy that must be stored to compress the device. This energy is approximately given by:

$$U_T = U_{\text{mech}} + U_{\text{elec}} = \frac{1}{2} k (g_{\text{c}})^2 + \frac{1}{2} \frac{\epsilon_0 \kappa w^2}{(g_0 - g_{\text{c}})} V^2 \tag{S8}$$

where U_{elec} is the electrical energy and U_{mech} is the mechanical energy. As our mechanics is dominated by the polymer layer and the electrodes are only electrically coupled through their overlap, this energy scales linearly with the active device area. Using our fitted device parameters and demonstrated dimensions (200 nm × 200 nm active area), we project $U_{\text{elec}} \sim 2$ fJ and $U_{\text{mech}} \sim 0.1$ fJ. Improvements are expected through further scaling of the active device area and materials engineering.

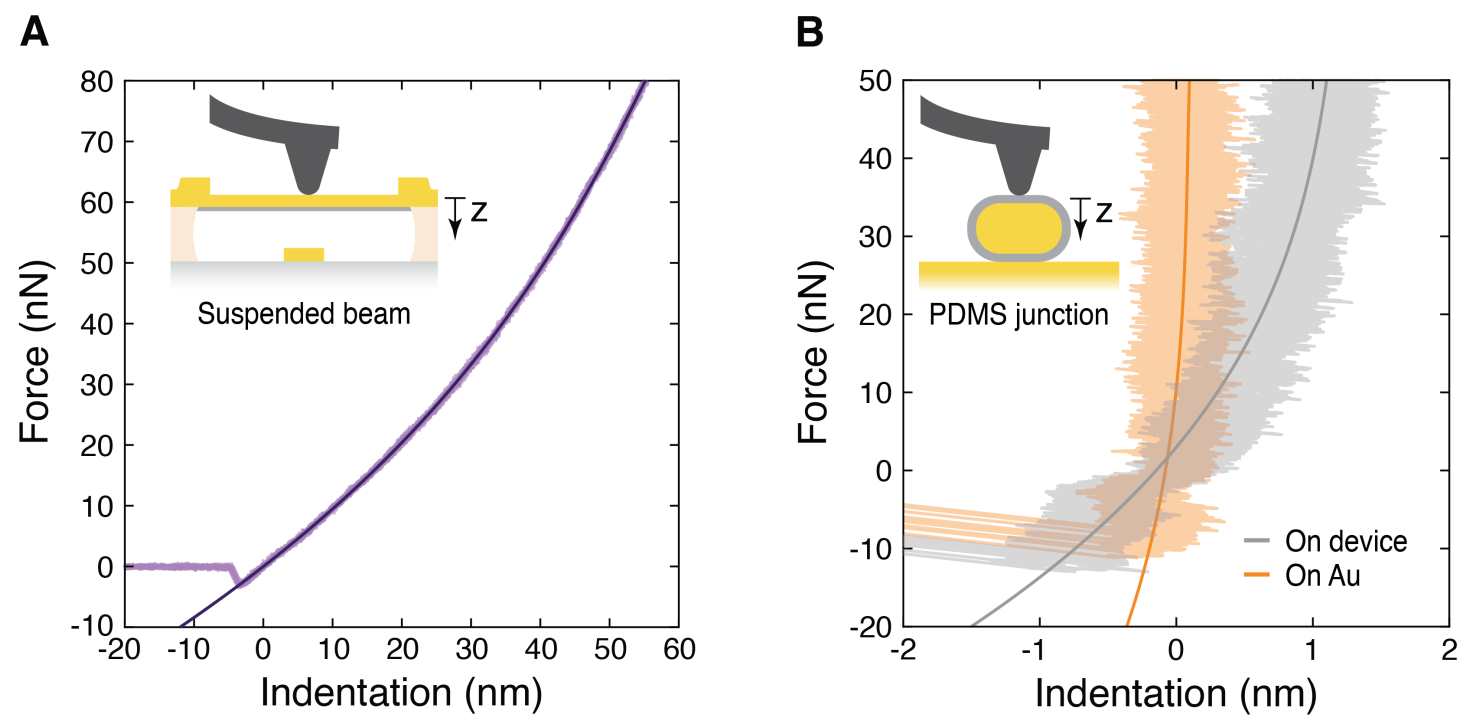

Fig. S1. Device characterization with force spectroscopy.

(A), Ten sequential force-indentation curves measured by pressing an AFM tip against a suspended top electrode beam prior to its collapse to form the PDMS junction, as shown in the inset schematic. **(B)**, Same measurement where AFM tip is pressed either against the PDMS junction as shown in the inset schematic, or against a bare Au electrode. The additional indentation in the device region represents the mechanical response of the polymer spacer corresponding to an elastic modulus of 10 MPa for ~ 2 nm-thick PDMS. The suspended beam is substantially softer than the PDMS layer, measured to have a spring constant of < 3 nN/nm compared to ~ 50 nN/nm of the PDMS. This suggests that the device mechanics are dominated by the PDMS and not by the top electrode following junction formation.

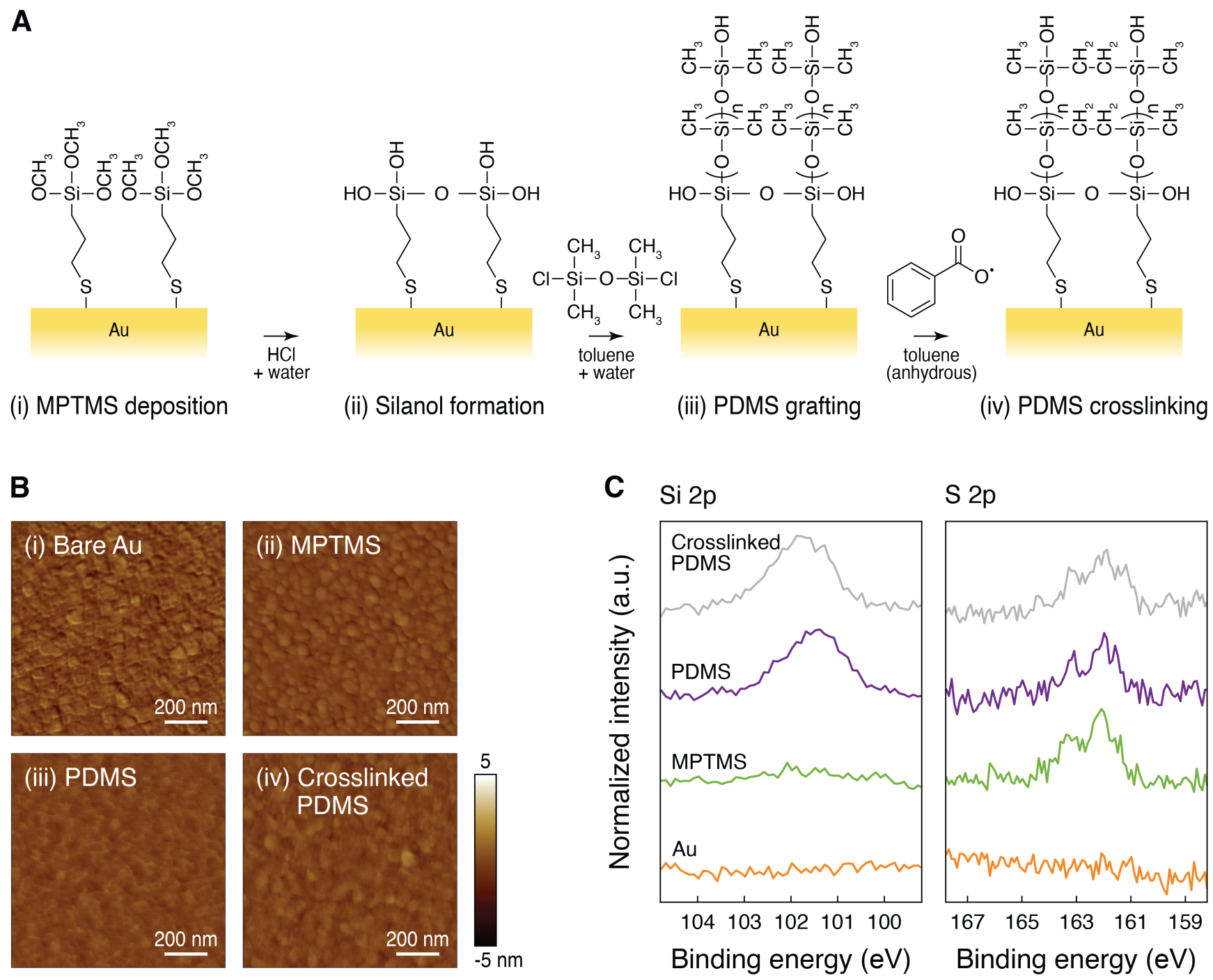

Fig. S2. Growth and characterization of PDMS nanometer-thin film.

(A) PDMS is grown through surface-initiated polymerization. First, a monolayer of (3-mercaptopropyl)trimethoxysilane (MPTMS) is assembled on the surface. The sample is placed in hydrochloric acid to hydrolyze the methoxysilanes to form reactive silanol groups. PDMS is then polymerized from this terminal group using 1,3-dichlorotetramethyldisiloxane (DCTMDS). The PDMS film is then crosslinked using benzoyl peroxide. **(B)** AFM surface topography of the sample during various stages of the PDMS growth process. Root-mean-square surface roughness values are 0.58 nm, 0.36 nm, 0.28 nm, and 0.36 nm, respectively for (i)-(iv), as calculated over a 1 µm × 1 µm area. **(C)** XPS spectra (Si 2p and S 2p peaks) of the sample surface during the same processing stages.

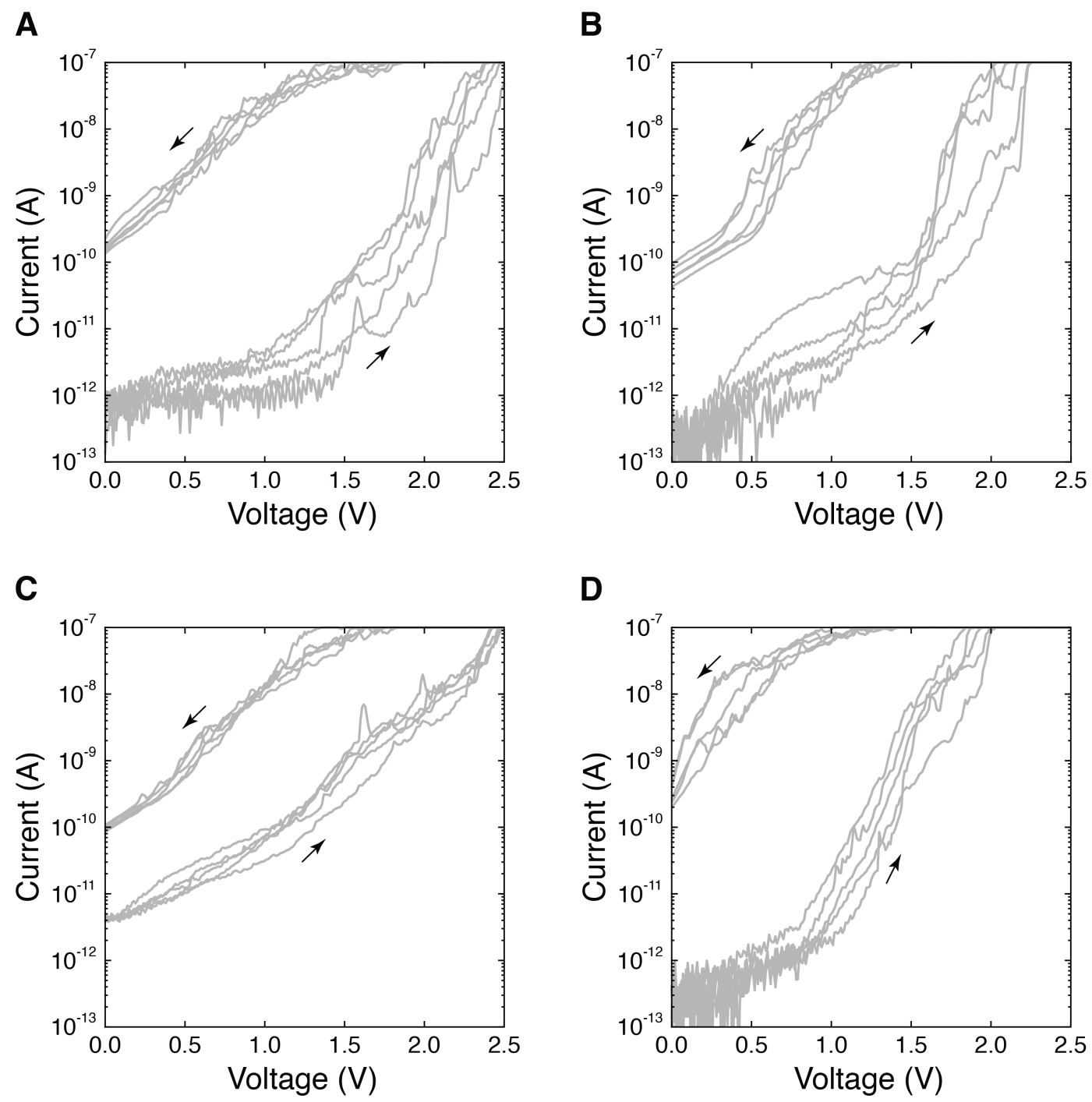

Fig. S3. Device performance across samples.
(A–D) *I–V* characteristics of four additional devices, structurally identical to the device in Fig. 1E, and independently processed on four substrates. Each panel shows five sequential runs.

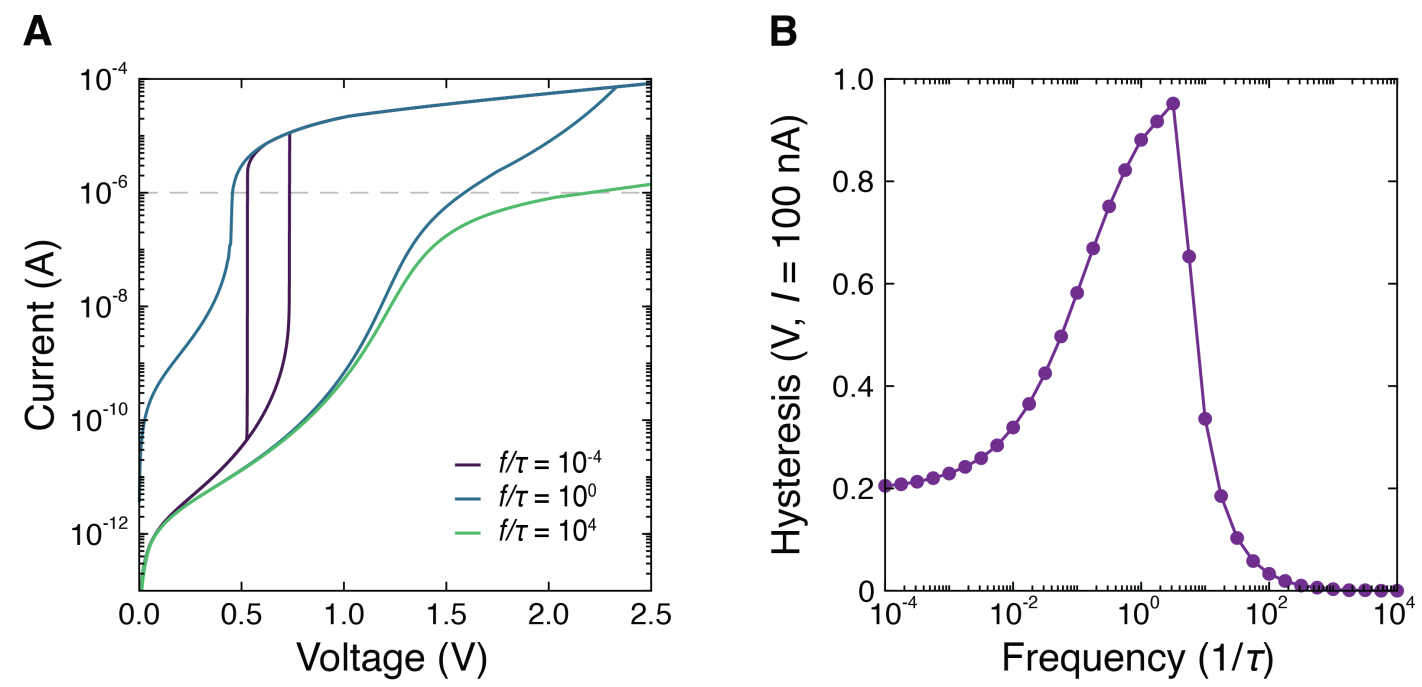

Fig. S4. Nanomechanical hysteresis.

(A) Modeled *I*–*V* characteristics at a frequency below, equal to and above the viscoelastic time constant (τ). Modeling parameters include: g_0 = 2.5 nm, g_{c1} = 1 nm, g_{c2} = 0.5 nm, E_1 = 15 MPa, E_2 = 20 MPa. **(B)** Voltage hysteresis at 100 nA (shown by the dotted line in (A)) as a function of frequency. Low-frequency measurements show hysteresis due to pull-in instability, which is enhanced by viscoelasticity, before disappearing at high frequency.

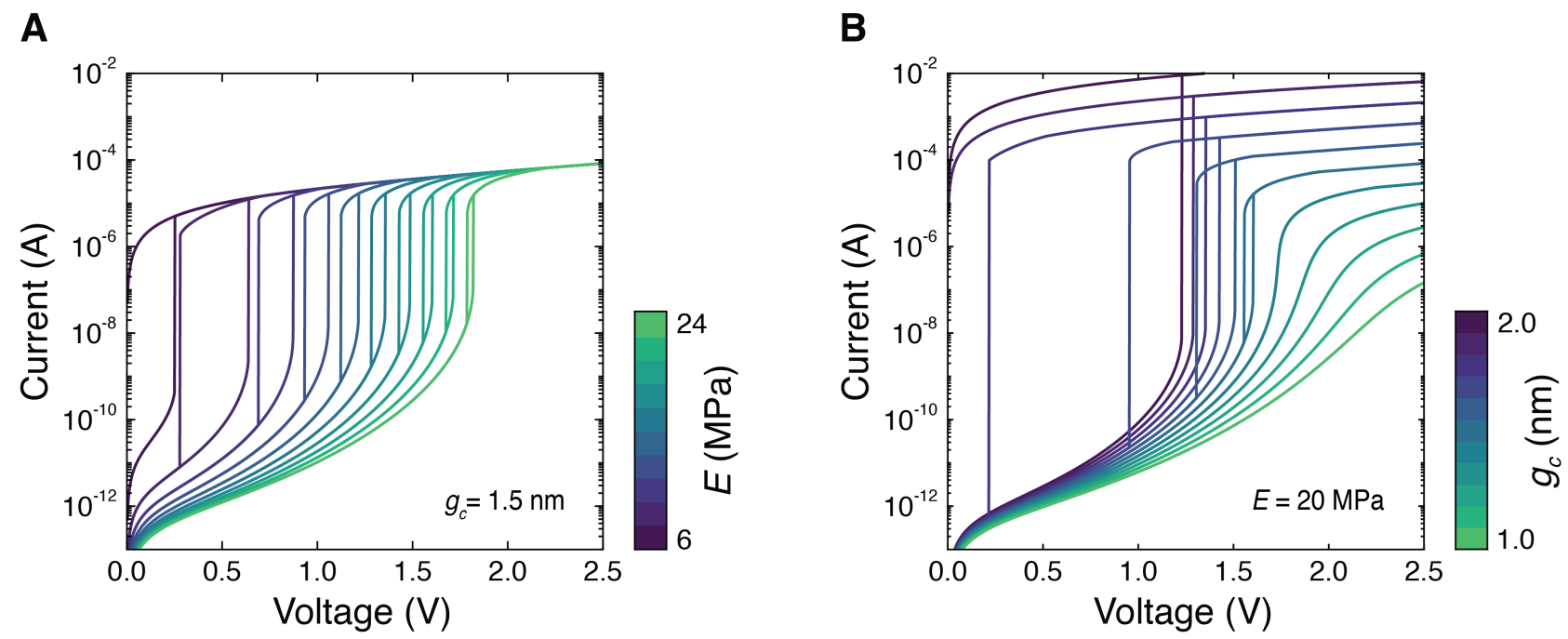

Fig. S5. Device engineering through mechanical response of the polymer spacer.

Simulated device performance as a function of **(A)** the elastic modulus (E) of the polymer spacer and **(B)** the thickness of the compressible portion of the gap (g_c). Decreasing the modulus lowers the voltage required to actuate the device and increases hysteresis. In addition, for a given modulus, increasing the compressible gap increases the hysteresis. For simplicity, this plot uses an elastic model with g_0 = 2.5 nm, not taking into account viscoelasticity.

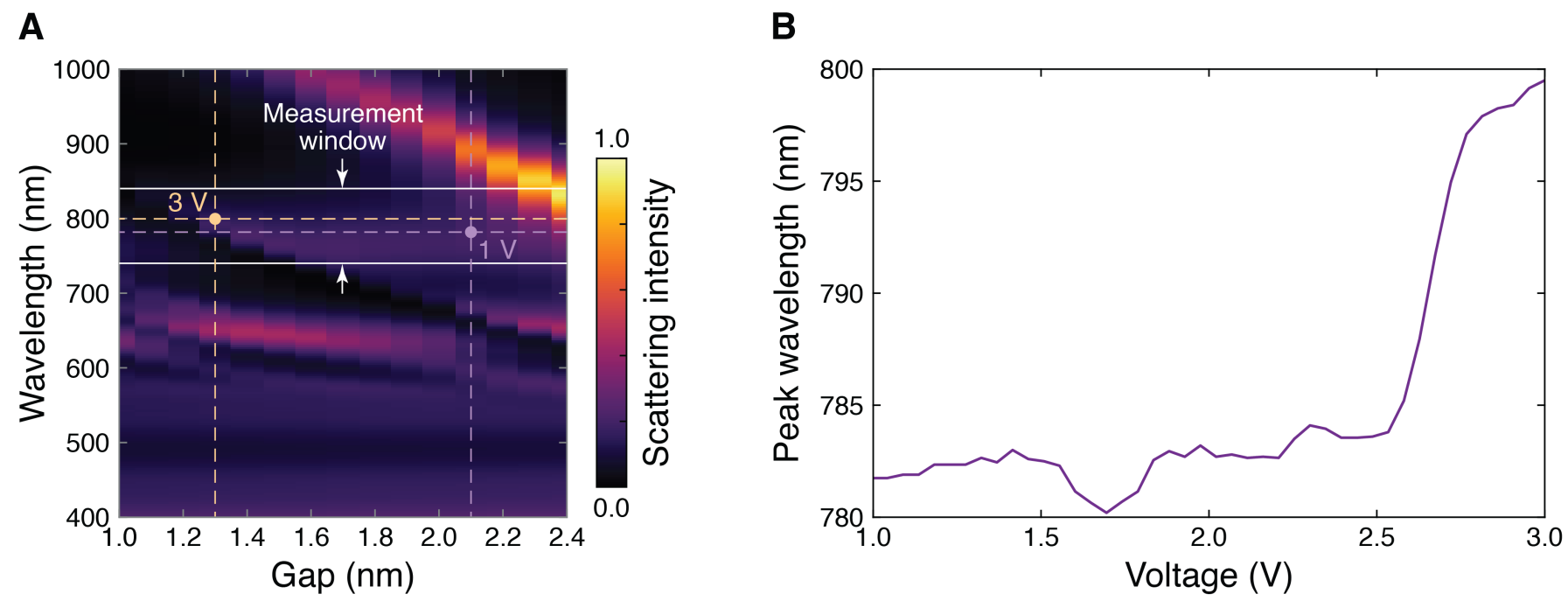

Fig. S6. Optical probing of the mechanical reconfiguration.

(A) Simulated dark-field scattering of the device with varying gaps. The experimental measurement window is overlaid. The dotted lines annotate the peak scattering wavelengths measured experimentally at 1 V (purple) and 3 V (orange), and the corresponding simulated gap sizes. The experimentally measured shift in the scattering wavelength is shown in **(B)**. This 18 nm shift when compared to the simulated behavior corresponds to ~ 0.8 nm decrease in the gap.

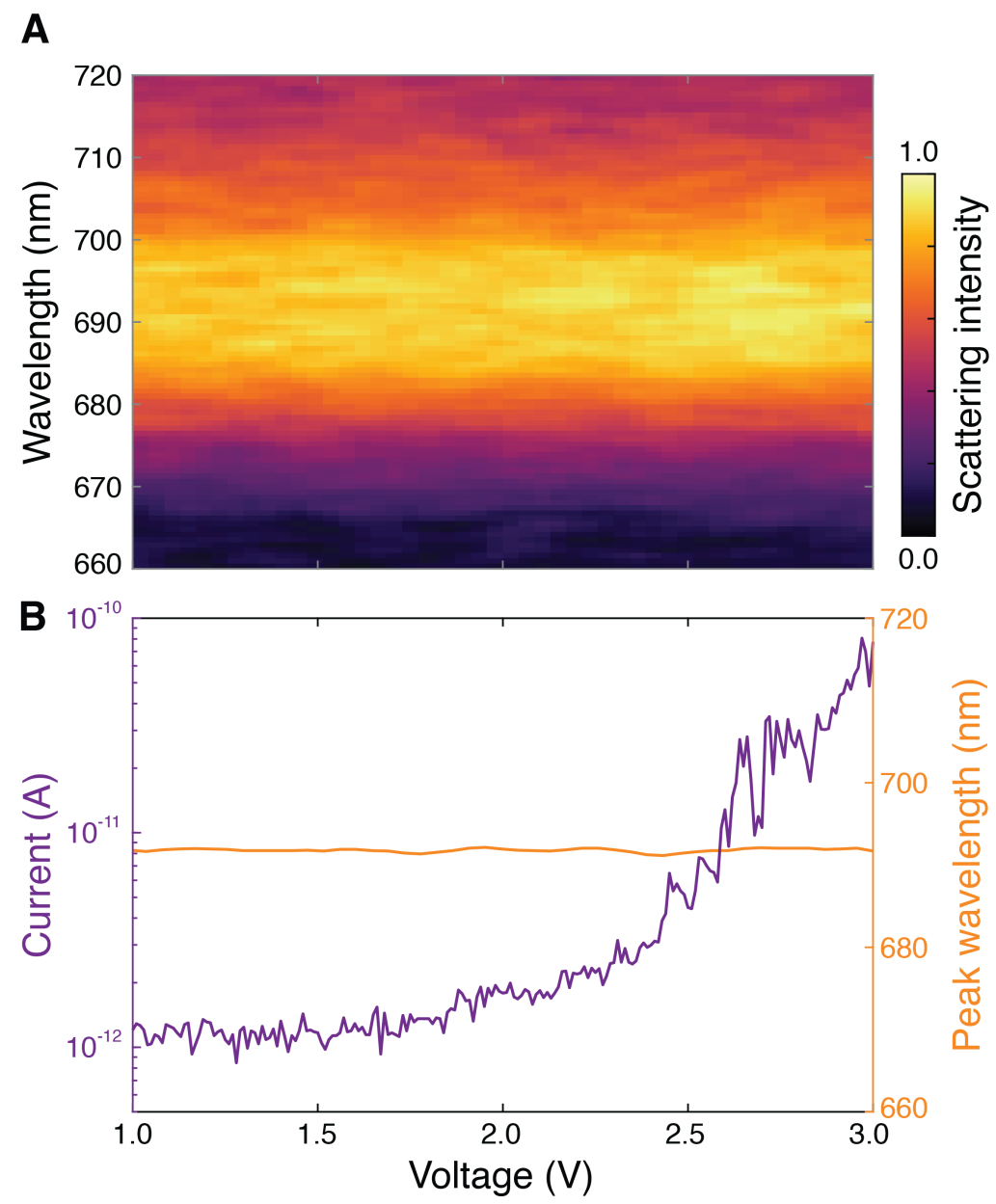

Fig. S7. Optical probing of a rigid device.

(A) Dark-field scattering spectrum as a function of applied voltage for a device fabricated with a ~ 1.2 nm Al_2O_3 gap. **(B)** The peak scattering wavelength as a function of applied voltage and the corresponding current measured through the device is shown. No shift in the optical response is observed.

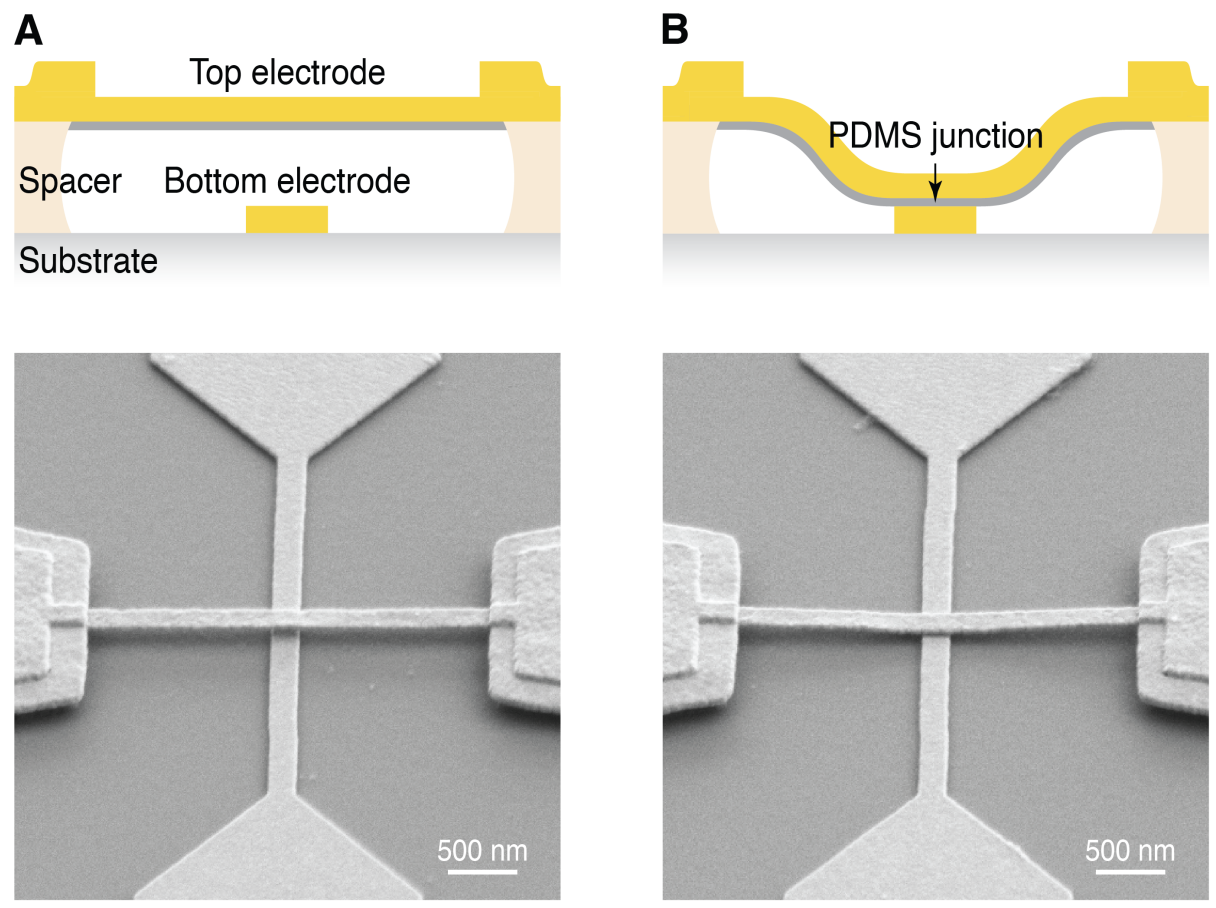

Fig. S8. Device formation through capillary collapse.

(A) Schematic and SEM image of the device scaffold, with the top electrode suspended to allow for the PDMS growth on the electrode surface. **(B)** Schematic and SEM image of the device following the PDMS growth and junction formation through capillary collapse, where the top and bottom electrodes are separated by the PDMS layer.

Table S1. Fitted device parameters for the device in Fig. 1E–F. The experimental data are fitted as described in supplementary text S1.

Parameter	Description	Value
$\boldsymbol{k_1}$ ($\boldsymbol{E_1}$)	Instantaneous spring constant (modulus)	840 nN/nm (23 MPa)
$\boldsymbol{k_2}$ ($\boldsymbol{E_2}$)	Viscoelastic spring constant (modulus)	230 nN/nm (6.2 MPa)
$\boldsymbol{g_0}$	Nominal gap	2.6 nm
$\boldsymbol{g_{c1}}$	Instantaneous compressible gap	0.42 nm
$\boldsymbol{g_{c2}}$	Viscoelastic compressible gap	0.65 nm
$\boldsymbol{\tau}$	Characteristic time constant	4.9 s

Table S2. Comparison with other artificial neuron technologies.

Reference	Technology	Area	Characteristic timescale	Required components‡	Demonstrated (projected) energy per firing event
Pickett et al. 2013 (*66*)	Mott memristor	0.01 μm^{2*} + discrete components	10-100 μs	2M, 3R, 3C	(100 fJ)
Tuma et al. 2016 (*67*)	Phase change neuron	> 0.5 μm^{2†}	10-100 μs	1M, 17T	> 5 pJ
Yi et al. 2018 (*43*)	VO_2 memristor	0.0025 μm^{2*} + discrete components	10-100 μs	2M, 2R, 2C	(2 fJ)
Chen et al. 2019 (*68*)	MXene memristor	Not reported	10 ms	1M	(20 nJ)
Dev et al. 2020 (*69*)	MoS_2 threshold switch	4 μm^{2*} + discrete components	10 ms	1M, 2R, 1C	Not reported
Liang et al. 2020 (*70*)	STT-MRAM	26 μm^{2†}	10 μs	2STT, 11T	Not reported
Han et al. 2021 (*71*)	SONOS transistor	0.25 μm^{2*}	10 ms	1T	30 pJ (0.7 pJ)
Saha et al. 2022 (*72*)	CMOS with NEMS neuron	896.2 μm^{2†}	1 μs	2N, 9T, 2C	46.7 fJ
Sarkar et al. 2022 (*73*)	Electrochemical device	1500 μm^{2*} + discrete components	1-100 ms	4ECT, 3R, 1C	57 nJ
Milozzi et al. 2024 (*74*)	Ag memristor	0.4 μm^{2†}	100 μs-100 ms	1M, 1T	40 pJ (40 fJ)
Pazos et al. 2025 (*75*)	MOSFET with impact ionization	0.2 μm^{2*}	1 μs-10 ms	1T	415 pJ (75 pJ)
Zhao et al. 2025 (*76*)	Transistor driven by diffusive memristor	4 μm^{2*}	1-100 ms	1M, 1T, 1R	6 pJ (1 aJ)
**This work**	**Viscoelastic neuron**	**0.04 μm^{2*}**	**1 s**	**1N**	**54 nJ (~ 2 fJ)**

*Active device area, not including peripheral wires, pads and circuitry.

†Circuit area needed per artificial neuron.

‡C represents capacitor, ECT represents electrochemical transistor, M represents memristor, N represents NEMS device, R represents resistor, STT represents spin-transfer torque, T represents transistor.

REFERENCES

1. H. Yasuda, P. R. Buskohl, A. Gillman, T. D. Murphey, S. Stepney, R. A. Vaia, J. R. Raney, Mechanical computing. *Nature* **598**, 39–48 (2021).

2. C. Kaspar, B. J. Ravoo, W. G. van der Wiel, S. V. Wegner, W. H. P. Pernice, The rise of intelligent matter. *Nature* **594**, 345–355 (2021).

3. L. G. Wright, T. Onodera, M. M. Stein, T. Wang, D. T. Schachter, Z. Hu, P. L. McMahon, Deep physical neural networks trained with backpropagation. *Nature* **601**, 549–555 (2022).

4. J. Byun, A. Pal, J. Ko, M. Sitti, Integrated mechanical computing for autonomous soft machines. *Nat. Commun.* **15**, 2933 (2024).

5. C. El Helou, B. Grossmann, C. E. Tabor, P. R. Buskohl, R. L. Harne, Mechanical integrated circuit materials. *Nature* **608**, 699–703 (2022).

6. T. Chen, M. Pauly, P. M. Reis, A reprogrammable mechanical metamaterial with stable memory. *Nature* **589**, 386–390 (2021).

7. T. Mei, C. Q. Chen, In-memory mechanical computing. *Nat. Commun.* **14**, 5204 (2023).

8. R. H. Lee, E. A. B. Mulder, J. B. Hopkins, Mechanical neural networks: Architected materials that learn behaviors. *Sci. Robot.* **7**, eabq7278 (2022).

9. X. Guo, W. Yang, X. Xiong, Z. Wang, X. Zou, MEMS reservoir computing system with stiffness modulation for multi-scene data processing at the edge. *Microsyst. Nanoeng.* **10**, 84 (2024).

10. J. Sun, W. Yang, T. Zheng, X. Xiong, Y. Liu, Z. Wang, Z. Li, X. Zou, Novel nondelay-based reservoir computing with a single micromechanical nonlinear resonator for high-efficiency information processing. *Microsyst. Nanoeng.* **7**, 83 (2021).

11. H. Nikfarjam, M. Megdadi, M. Okour, S. Pourkamali, F. Alsaleem, Energy efficient integrated MEMS neural network for simultaneous sensing and computing. *Commun. Eng.* **2**, 19 (2023).

12. A. Alù, A. F. Arrieta, E. Del Dottore, M. Dickey, S. Ferracin, R. Harne, H. Hauser, Q. He, J. B. Hopkins, L. P. Hyatt, S. Li, S. Mariani, B. Mazzolai, A. Mondini, A. Pal, D. J. Preston, A. Rajappan, J. R. Raney, P. M. Reis, S. A. Sarles, M. Sitti, U. K. Ubamanyu, M. van Hecke, K. W. Wang, Roadmap on embodying mechano-intelligence and computing in functional materials and structures. *Smart Mater. Struct.* **34**, 063501 (2025).

13. D. J. Preston, P. Rothemund, H. J. Jiang, M. P. Nemitz, J. Rawson, Z. Suo, G. M. Whitesides, Digital logic for soft devices. *Proc. Natl. Acad. Sci. U.S.A.* **116**, 7750–7759 (2019).

14. Y. Song, R. M. Panas, S. Chizari, L. A. Shaw, J. A. Jackson, J. B. Hopkins, A. J. Pascall, Additively manufacturable micro-mechanical logic gates. *Nat. Commun.* **10**, 882 (2019).

15. H. Yasuda, T. Tachi, M. Lee, J. Yang, Origami-based tunable truss structures for non-volatile mechanical memory operation. *Nat. Commun.* **8**, 962 (2017).

16. T. Mei, Z. Meng, K. Zhao, C. Q. Chen, A mechanical metamaterial with reprogrammable logical functions. *Nat. Commun.* **12**, 7234 (2021).

17. C. El Helou, P. R. Buskohl, C. E. Tabor, R. L. Harne, Digital logic gates in soft, conductive mechanical metamaterials. *Nat. Commun.* **12**, 1633 (2021).

18. K. Bertoldi, V. Vitelli, J. Christensen, M. van Hecke, Flexible mechanical metamaterials. *Nat. Rev. Mater.* **2**, 17066 (2017).

19. Q. He, S. Ferracin, J. R. Raney, Programmable responsive metamaterials for mechanical computing and robotics. *Nat. Comput. Sci.* **4**, 567–573 (2024).

20. V. A. Baulin, A. Giacometti, D. A. Fedosov, S. Ebbens, N. R. Varela-Rosales, N. Feliu, M. Chowdhury, M. Hu, R. Füchslin, M. Dijkstra, M. Mussel, R. van Roij, D. Xie, V. Tzanov, M. Zu, S. Hidalgo-Caballero, Y. Yuan, L. Cocconi, C.-M. Ghim, C. Cottin-Bizonne, M. C. Miguel, M. J. Esplandiu, J. Simmchen, W. J. Parak, M. Werner, G. Gompper, M. M. Hanczyc, Intelligent soft matter: Towards embodied intelligence. *Soft Matter* **21**, 4129–4145 (2025).

21. S. Li, X. Mao, Training all-mechanical neural networks for task learning through in situ backpropagation. *Nat. Commun.* **15**, 10528 (2024).

22. L. Wang, P. Zhang, Z. Liu, Z. Wang, R. Yang, On-chip mechanical computing: Status, challenges, and opportunities. *Chip* **2**, 100038 (2023).

23. Y.-B. Lee, M.-H. Kang, P.-K. Choi, S.-H. Kim, T.-S. Kim, S.-Y. Lee, J.-B. Yoon, Sub-10 fJ/bit radiation-hard nanoelectromechanical non-volatile memory. *Nat. Commun.* **14**, 460 (2023).

24. J. Han, Z. Nelson, M. R. Chua, T. M. Swager, F. Niroui, J. H. Lang, V. Bulović, Molecular platform for fast low-voltage nanoelectromechanical switching. *Nano Lett.* **21**, 10244–10251 (2021).

25. B. Osoba, B. Saha, L. Dougherty, J. Edgington, C. Qian, F. Niroui, J. H. Lang, V. Bulović, J. Wu, T.-J. K. Liu, Sub-50 mV NEM relay operation enabled by self-assembled molecular coating, in *2016 IEEE International Electron Devices Meeting (IEDM)* (IEEE, 2016).

26. M. A. A. Hafiz, L. Kosuru, M. I. Younis, Microelectromechanical reprogrammable logic device. *Nat. Commun.* **7**, 11137 (2016).

27. T.-H. Lee, S. Bhunia, M. Mehregany, Electromechanical computing at 500°C with silicon carbide. *Science* **329**, 1316–1318 (2010).

28. S. Rana, J. Mouro, S. J. Bleiker, J. D. Reynolds, H. M. H. Chong, F. Niklaus, D. Pamunuwa, Nanoelectromechanical relay without pull-in instability for high-temperature non-volatile memory. *Nat. Commun.* **11**, 1181 (2020).

29. R. Maboudian, R. T. Howe, Critical review: Adhesion in surface micromechanical structures. *J. Vac. Sci. Technol. B* **15**, 1–20 (1997).

30. S. O. Spector, P. F. Satterthwaite, M. Conte, T. Hsieh, E. O. Bobylev, K. Dunn, W. Zhu, J. Sim, J. A. Johnson, F. Niroui, Self-assembled contacts for high-yield molecular devices. *Nat. Nanotechnol.*, https://doi.org/10.1038/s41565-026-02227-9 (2026).

31. A. Vilan, Analyzing molecular current-voltage characteristics with the Simmons tunneling model: Scaling and linearization. *J. Phys. Chem. C* **111**, 4431–4444 (2007).

32. J. G. Simmons, Generalized formula for the electric tunnel effect between similar electrodes separated by a thin insulating film. *J. Appl. Phys.* **34**, 1793–1803 (1963).

33. W. Chen, S. Zhang, Q. Deng, H. Xu, Probing of sub-picometer vertical differential resolutions using cavity plasmons. *Nat. Commun.* **9**, 801 (2018).

34. R. T. Hill, J. J. Mock, A. Hucknall, S. D. Wolter, N. M. Jokerst, D. R. Smith, A. Chilkoti, Plasmon ruler with angstrom length resolution. *ACS Nano* **6**, 9237–9246 (2012).

35. S. D. Senturia, *Microsystem Design* (Springer, 2001).

36. J. Israelachvili, *Intermolecular and Surface Forces* (Elsevier, ed. 3, 2011).

37. I. M. Ward, J. Sweeney, *Mechanical Properties of Solid Polymers* (Wiley, ed. 3, 2012).

38. W. Xu, N. Chahine, T. Sulchek, Extreme hardening of PDMS thin films due to high compressive strain and confined thickness. *Langmuir* **27**, 8470–8477 (2011).

39. E. R. Kandel, J. D. Koester, S. H. Mack, S. A. Siegelbaum, *Principles of Neural Science* (McGraw Hill, ed. 6, 2021).

40. K. Roy, A. Jaiswal, P. Panda, Towards spike-based machine intelligence with neuromorphic computing. *Nature* **575**, 607–617 (2019).

41. G. Indiveri, S.-C. Liu, Memory and information processing in neuromorphic systems. *Proc. IEEE* **103**, 1379–1397 (2015).

42. L. Porath, J. Huang, N. Ramlawi, M. Derkaloustian, R. H. Ewoldt, C. M. Evans, Relaxation of vitrimers with kinetically distinct mixed dynamic bonds. *Macromolecules* **55**, 4450–4458 (2022).

43. W. Yi, K. K. Tsang, S. K. Lam, X. Bai, J. A. Crowell, E. A. Flores, Biological plausibility and stochasticity in scalable VO_2 active memristor neurons. *Nat. Commun.* **9**, 4661 (2018).

44. W. B. Levy, V. G. Calvert, Communication consumes 35 times more energy than computation in the human cortex, but both costs are needed to predict synapse number. *Proc. Natl. Acad. Sci. U.S.A.* **118**, e2008173118 (2021).

45. R. Benavides-Piccione, L. Blazquez-Llorca, A. Kastanauskaite, I. Fernaud-Espinosa, S. Tapia-González, J. DeFelipe, Key morphological features of human pyramidal neurons. *Cereb. Cortex* **34**, bhae180 (2024).

46. L. Luo, Architectures of neuronal circuits. *Science* **373**, eabg7285 (2021).

47. J. Borghetti, G. S. Snider, P. J. Kuekes, J. J. Yang, D. R. Stewart, R. S. Williams, 'Memristive' switches enable 'stateful' logic operations via material implication. *Nature* **464**, 873–876 (2010).

48. T. Emmerich, Y. Teng, N. Ronceray, E. Lopriore, R. Chiesa, A. Chernev, V. Artemov, M. Di Ventra, A. Kis, A. Radenovic, Nanofluidic logic with mechano–ionic memristive switches. *Nat. Electron.* **7**, 271–278 (2024).

49. R. Rubin, L. F. Abbott, H. Sompolinsky, Balanced excitation and inhibition are required for high-capacity, noise-robust neuronal selectivity. *Proc. Natl. Acad. Sci. U.S.A.* **114**, E9366–E9375 (2017).

50. M. M. Caruso, D. A. Davis, Q. Shen, S. A. Odom, N. R. Sottos, S. R. White, J. S. Moore, Mechanically-induced chemical changes in polymeric materials. *Chem. Rev.* **109**, 5755–5798 (2009).

51. S. P. Selvin, M. Esfandyarpour, A. Ji, Y. J. Lee, C. Yule, J.-H. Song, M. Taghinejad, M. L. Brongersma, Acoustic wave modulation of gap plasmon cavities. *Science* **389**, 516–520 (2025).

52. I. Piwoński, J. Grobelny, M. Cichomski, G. Celichowski, J. Rogowski, Investigation of 3-mercaptopropyltrimethoxysilane self-assembled monolayers on Au (111) surface. *Appl. Surf. Sci.* **242**, 147–153 (2005).

53. J. Liu, Y. Sun, X. Zhou, X. Li, M. Kappl, W. Steffen, H.-J. Butt, One-step synthesis of a durable and liquid-repellent poly(dimethylsiloxane) coating. *Adv. Mater.* **33**, 2100237 (2021).

54. A. M. Bueche, The curing of silicone rubber with benzoyl peroxide. *J. Polym. Sci.* **15**, 105–120 (1955).

55. S. J. Harley, E. A. Glascoe, R. S. Maxwell, Thermodynamic study on dynamic water vapor sorption in Sylgard-184. *J. Phys. Chem. B* **116**, 14183–14190 (2012).

56. S. Hu, E. Elliott, A. Sánchez-Iglesias, J. Huang, C. Guo, Y. Hou, M. Kamp, E. S. A. Goerlitzer, K. Bedingfield, B. de Nijs, J. Peng, A. Demetriadou, L. M. Liz-Marzán, J. J. Baumberg, Full control of plasmonic nanocavities using gold decahedra-on-mirror constructs with monodisperse facets. *Adv. Sci.* **10**, 2207178 (2023).

57. H. Krupp, W. Schnabel, G. Walter, The Lifshitz-van der Waals constant: Computation of the Lifshitz-van der Waals constant on the basis of optical data. *J. Colloid Interface Sci.* **39**, 421–423 (1972).

58. J. E. Mark, Ed., *Polymer Data Handbook* (Oxford Univ. Press, ed. 2, 2009).

59. M. Rubinstein, R. H. Colby, *Polymer Physics* (Oxford Univ. Press, 2003).

60. H. Dal, K. Açıkgöz, Y. Badienia, On the performance of isotropic hyperelastic constitutive models for rubber-like materials: A state of the art review. *Appl. Mech. Rev.* **73**, 020802 (2021).

61. H. He, Q. Zhang, Y. Zhang, J. Chen, L. Zhang, F. Li, A comparative study of 85 hyperelastic constitutive models for both unfilled rubber and highly filled rubber nanocomposite material. *Nano Mater. Sci.* **4**, 64–82 (2022).

62. W. Wang, T. Lee, M. A. Reed, Mechanism of electron conduction in self-assembled alkanethiol monolayer devices. *Phys. Rev. B* **68**, 035416 (2003).

63. J. R. Niskala, W. C. Rice, R. C. Bruce, T. J. Merkel, F. Tsui, W. You, Tunneling characteristics of Au–alkanedithiol–Au junctions formed via nanotransfer printing (nTP). *J. Am. Chem. Soc.* **134**, 12072–12082 (2012).

64. P. B. Johnson, R. W. Christy, Optical constants of the noble metals. *Phys. Rev. B* **6**, 4370–4379 (1972).

65. F. Schneider, J. Draheim, R. Kamberger, U. Wallrabe, Process and material properties of polydimethylsiloxane (PDMS) for optical MEMS. *Sens. Actuators A: Phys.* **151**, 95–99 (2009).

66. M. D. Pickett, G. Medeiros-Ribeiro, R. S. Williams, A scalable neuristor built with Mott memristors. *Nat. Mater.* **12**, 114–117 (2013).

67. T. Tuma, A. Pantazi, M. Le Gallo, A. Sebastian, E. Eleftheriou, Stochastic phase-change neurons. *Nat. Nanotechnol.* **11**, 693–699 (2016).

68. Y. Chen, Y. Wang, Y. Luo, X. Liu, Y. Wang, F. Gao, J. Xu, E. Hu, S. Samanta, X. Wan, X. Lian, J. Xiao, Y. Tong, Realization of artificial neuron using MXene bi-directional threshold switching memristors. *IEEE Electron Device Lett.* **40**, 1686–1689 (2019).

69. D. Dev, A. Krishnaprasad, M. S. Shawkat, Z. He, S. Das, D. Fan, H.-S. Chung, Y. Jung, T. Roy, 2D MoS_2-based threshold switching memristor for artificial neuron. *IEEE Electron Device Lett.* **41**, 936–939 (2020).

70. F.-X. Liang, P. Sahu, M.-H. Wu, J.-H. Wei, S.-S. Sheu, T.-H. Hou, Stochastic STT-MRAM spiking neuron circuit, in *2020 International Symposium on VLSI Technology, Systems and Applications (VLSI-TSA)* (IEEE, 2020), pp. 151–152.

71. J.-K. Han, J. Oh, G.-J. Yun, D. Yoo, M.-S. Kim, J.-M. Yu, S.-Y. Choi, Y.-K. Choi, Cointegration of single-transistor neurons and synapses by nanoscale CMOS fabrication for highly scalable neuromorphic hardware. *Sci. Adv.* **7**, eabg8836 (2021).

72. S. Saha, P. B. Kanakya, M. Goel, M. S. Baghini, V. R. Rao, Energy efficient LIF neuron circuit using hybrid CMOS-NEMS in 65 nm CMOS technology, in *2022 IEEE 35th International Conference on Micro Electro Mechanical Systems (MEMS)* (IEEE, 2022), pp. 17–20.

73. T. Sarkar, K. Lieberth, A. Pavlou, T. Frank, V. Mailaender, I. McCulloch, P. W. M. Blom, F. Torricelli, P. Gkoupidenis, An organic artificial spiking neuron for in situ neuromorphic sensing and biointerfacing. *Nat. Electron.* **5**, 774–783 (2022).

74. A. Milozzi, S. Ricci, D. Ielmini, Memristive tonotopic mapping with volatile resistive switching memory devices. *Nat. Commun.* **15**, 2812 (2024).

75. S. Pazos, K. Zhu, M. A. Villena, O. Alharbi, W. Zheng, Y. Shen, Y. Yuan, Y. Ping, M. Lanza, Synaptic and neural behaviours in a standard silicon transistor. *Nature* **640**, 69–76 (2025).

76. R. Zhao, T. Wang, T. Moon, Y. Xu, J. Zhao, P. Sud, S. J. Kim, H.-T. Liao, Y. Zhuo, R. Midya, S. Asapu, D. Gao, Z. Rong, Q. Qiu, C. Bowers, K. Mahalingam, S. Ganguli, A. K. Roy, Q. Wu, J.-W. Han, R. S. Williams, Y. Chen, J. J. Yang, A spiking artificial neuron based on one diffusive memristor, one transistor and one resistor. *Nat. Electron.* **8**, 1211–1221 (2025).
